# Oxidative Stress, Inflammation, and Cellular Senescence in Neuropathic Pain: Mechanistic Crosstalk

**DOI:** 10.3390/antiox14101166

**Published:** 2025-09-25

**Authors:** Bojan Stojanovic, Ivana Milivojcevic Bevc, Milica Dimitrijevic Stojanovic, Bojana S. Stojanovic, Tatjana Lazarevic, Marko Spasic, Marko Petrovic, Ivana Stefanovic, Marina Markovic, Jelena Nesic, Danijela Jovanovic, Miodrag Peulic, Ana Azanjac Arsic, Ana Lukovic, Nikola Mirkovic, Stevan Eric, Nenad Zornic

**Affiliations:** 1 Department of Surgery, Faculty of Medical Sciences, University of Kragujevac, 34000 Kragujevac, Serbia; bojanstojanovic@medf.kg.ac.rs (B.S.);; 2 Center for Molecular Medicine and Stem Cell Research, Faculty of Medical Sciences, University of Kragujevac, 34000 Kragujevac, Serbia; 3 City Medical Emergency Department, 11000 Belgrade, Serbia; 4 Department of Pathology, Faculty of Medical Sciences, University of Kragujevac, 34000 Kragujevac, Serbia; 5 Department of Pathophysiology, Faculty of Medical Sciences, University of Kragujevac, 34000 Kragujevac, Serbia; 6 Department of Internal Medicine, Faculty of Medical Sciences, University of Kragujevac, 34000 Kragujevac, Serbia; 7 Department of Neurology, Faculty of Medical Sciences, University of Kragujevac, 34000 Kragujevac, Serbia

**Keywords:** neuropathic pain, oxidative stress, mitochondrial dysfunction, cellular senescence, Nrf2, neuroinflammation

## Abstract

Neuropathic pain is a chronic condition driven by intertwined mechanisms of oxidative stress, inflammation, and cellular senescence. Nerve injury and metabolic stress elevate reactive oxygen and nitrogen species, disrupt mitochondrial function, and activate the DNA-damage response, which stabilizes p53 and induces p16/p21-mediated cell-cycle arrest. These events promote a senescence-associated secretory phenotype (SASP) rich in cytokines, chemokines, and prostanoids that amplify neuroimmune signaling. In the spinal dorsal horn and dorsal root ganglia, microglia and astroglia respond to redox imbalance and danger cues by engaging NF-κB and MAPK pathways, increasing COX-2–dependent prostaglandin synthesis, and releasing mediators such as IL-1β and BDNF that enhance synaptic transmission and reduce inhibitory tone through KCC2 dysfunction. At the periphery, persistent immune-glial cross-talk lowers activation thresholds of nociceptors and sustains ectopic firing, while impaired autophagy and mitophagy further exacerbate mitochondrial dysfunction and ROS production. Collectively, these processes establish a feed-forward loop in which redox imbalance triggers senescence programs and SASP, SASP perpetuates neuroinflammation, and neuroinflammation maintains central sensitization—thereby consolidating a self-sustaining redox–senescence–inflammatory circuit underlying neuropathic pain chronicity.

## 1. Introduction

Neuropathic pain persists through tightly interwoven mechanisms of oxidative stress, inflammation, and cellular senescence, with Nrf2 functioning as an integrative hub that links these pathways to the initiation and maintenance of pain. Yet, despite rapid advances across redox biology, glial neuroimmunology, and senescence research, the field lacks a coherent framework that explains how mitochondrial dysfunction and ROS/RNS trigger DNA-damage responses and p16/p21-driven arrest, how the senescence-associated secretory phenotype amplifies microglial and astroglial signaling (e.g., NF-κB/MAPK, COX-2, IL-1β, BDNF), and how these processes remodel nociceptive circuits from primary afferents to the dorsal horn to consolidate central sensitization. This review’s objective is to articulate an integrative, pathway-level model that maps the crosstalk among oxidative stress, inflammation, and senescence across relevant cell types (neurons, microglia, astroglia, immune cells) and etiologies of neuropathic pain, closing the knowledge gap by synthesizing and critically interpreting contemporary experimental, translational, and clinical findings into a unified mechanistic narrative.

## 2. Foundations of Pain: From Protective Mechanism to Pathological Persistence

Pain represents a multidimensional sensory and emotional experience that functions as an essential protective mechanism, alerting the organism to actual or potential tissue injury [1]. It arises through the activation of specialized peripheral receptors and neural pathways that convey noxious information to higher brain centers, where it is integrated with cognitive and emotional components [2,3]. The International Association for the Study of Pain defines it as an unpleasant sensory and emotional experience related to, or resembling that associated with, tissue damage, thereby acknowledging its occurrence in both the presence and absence of ongoing structural injury [4]. Pain may arise from peripheral nociceptor activation subsequent to tissue injury, from pathological alterations within the somatosensory nervous system, or from modified nociceptive processing in the absence of definitive injury or disease [5,6]. The first mechanism, which is usually linked to acute, well-localized pain, typically subsides as the underlying damage heals [6]. In contrast, injury or dysfunction of neural pathways can produce pain that persists independently of tissue recovery, reflecting maladaptive signaling within the nervous system [7]. A third pathway involves disturbances in nociceptive modulation, often manifesting in conditions such as fibromyalgia or migraine, where central sensitization dominates [8]. In specific contexts, pain is exacerbated by inflammatory processes wherein immune-mediated responses sensitize peripheral receptors and sustain nociceptive input [9]. These mechanisms are not mutually exclusive and often coexist in chronic pain conditions, resulting in intricate clinical presentations that require meticulous mechanistic evaluation to inform targeted therapeutic approaches.

### 2.1. Definitional Evolution and Conceptual Challenges in Neuropathic Pain

The concept of neuropathic pain has undergone significant refinement over the past decades as experts have sought to achieve greater diagnostic precision [10]. The earliest widely adopted definition by the International Association for the Study of Pain (IASP) described it as pain initiated or caused by a primary lesion or dysfunction of the nervous system [11]. Although widely used, this wording was criticized because the term “dysfunction” was seen by many as too broad and unclear for diagnosis [12]. With this definition, some long-term pain conditions without clear nerve damage, such as fibromyalgia or irritable bowel syndrome, where the brain processes pain differently, could be wrongly labeled as neuropathic [13]. In 2008, a revised definition was proposed, restricting neuropathic pain to that arising as a direct consequence of a lesion or disease affecting the somatosensory system [14]. This update, approved by the IASP in 2011, left out conditions where problems in the motor system cause pain and removed “dysfunction” as a diagnostic criterion because it cannot be reliably confirmed [15].

Although the new definition is clearer, it still has limits. A problem in the sensory nervous system is required, but it is not enough on its own, since only some people with nerve damage actually develop neuropathic pain [15]. The pain experience is more often the result of maladaptive peripheral and central neuroplastic changes—such as sensitization, altered inhibition, and aberrant signaling—triggered by the lesion rather than the lesion itself [7,16].

### 2.2. Clinical Symptomatology of Neuropathic Pain

Neuropathic pain presents with a complex constellation of sensory abnormalities that reflect both gain and loss of function within the somatosensory system [17]. Patients frequently describe the pain as burning, shooting, stabbing, or accompanied by tingling sensations, which may arise spontaneously without any external stimulus [17,18]. A hallmark feature is the paradoxical combination of hypersensitivity and sensory deficit within the same affected area [19]. Hypersensitivity can manifest as pain evoked by normally non-painful stimuli, as in allodynia, or as an exaggerated response to painful stimuli, as in hyperalgesia [10]. These positive sensory phenomena are often accompanied by dysesthesia, characterized by unpleasant abnormal sensations, and paresthesia, involving abnormal but typically non-painful sensations such as pins and needles or numbness, which can nonetheless be distressing [10,20]. Negative sensory signs, including reduced sensitivity or complete loss of sensation in the painful region, frequently coexist, reflecting the underlying neuronal damage [17,21]. In many cases, symptoms persist or recur long after the initial injury has healed, and the pain may be exacerbated by movement or touch [21]. The chronicity of neuropathic pain is sustained by aberrant neural signaling and maladaptive neuroplastic changes, which contribute to the persistent mismatch between sensory input and perception, ultimately impairing physical function and psychological well-being [21].

### 2.3. Epidemiology of Neuropathic Pain

Estimating the true global burden of neuropathic pain remains challenging, largely due to the variability in diagnostic criteria and the absence of a universally accepted definition [22]. Population-based studies and systematic reviews suggest that chronic neuropathic pain affects a substantial proportion of adults, with prevalence rates ranging from approximately 3% to 17% depending on the population studied and the assessment tools employed [23]. Incidence estimates vary widely across specific syndromes: post-herpetic neuralgia and painful diabetic neuropathy represent some of the most common forms, while trigeminal and glossopharyngeal neuralgia occur less frequently [24]. The distribution of neuropathic pain is not uniform across demographic groups. Women appear disproportionately affected, accounting for over half of reported cases, and prevalence tends to rise with age, peaking in individuals between 50 and 64 years [25]. Occupational and environmental factors also influence disease distribution, with higher rates observed among manual laborers and residents of rural areas, suggesting that lifestyle, access to healthcare, and physical workload may contribute to risk [23].

### 2.4. Pathogenesis of Neuropathic Pain

**Figure 1:**
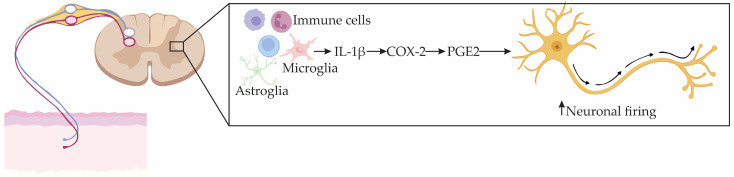
Neuroimmune IL-1β–COX-2–PGE2 pathway in spinal nociceptive sensitization. Immune cells, microglia, and astroglia release IL-1β, which triggers NF-κB and MAPK signaling, leading to COX-2 induction and prostaglandin E2 production. PGE2 engages E-type prostanoid receptors on neurons to increase neuronal firing and synaptic transmission, sustaining central sensitization and chronic pain.

Neuropathic pain emerges from a sustained neuroimmune dialog that reshapes excitability in peripheral and central pathways [26,27,28]. The increase in pro-inflammatory cytokines, particularly interleukin-1β (IL-1β), released by immune cells, microglia, and astroglia in the spinal cord is a key initiating event [29,30]. Through IL-1 receptor signaling, IL-1β activates nuclear factor kappa-light-chain-enhancer of activated B cells (NF-κB) and mitogen-activated protein kinase (MAPK) pathways, thereby amplifying the transcription of inflammatory genes and priming cyclo-oxygenase-2 (COX-2) induction [30]. COX-2 then drives prostanoid synthesis, particularly prostaglandin E2 (PGE2), which engages E-type prostanoid (EP) receptors to enhance neuronal firing and synaptic transmission (Figure 1) [31]. In addition to maintaining nociceptive gain and lowering activation thresholds, this cytokine–eicosanoid axis also initiates a feed-forward inflammatory loop that supports chronicity and treatment refractoriness [32,33].

At the periphery, nerve injury triggers a rapid, compartmentalized inflammatory response [34]. Schwann cells dedifferentiate and initiate myelin clearance at the lesion, while resident mast cells degranulate, releasing histamine, serotonin, leukotrienes, and growth factors that immediately sensitize nociceptors and recruit neutrophils [9,35]. Neutrophils, in turn, secrete algogenic mediators and promote the influx of monocyte-derived macrophages and T cells [9]. Together with resident macrophages and Schwann cells, these populations phagocytose degenerated axons/myelin and secrete cytokines/chemokines that further heighten excitability [9]. Through their direct action on injury-upregulated receptors on nociceptors, T-helper (Th) subsets provide layered control with pro-inflammatory cytokines such as IL-1β, tumor necrosis factor-alpha (TNF-α), and interleukin-17 (IL-17), as well as counter-regulatory cytokines including interleukin-4 (IL-4) and interleukin-10 (IL-10), thereby shaping continuous input to the central nervous system [9,35,36].

Altered impulse traffic from injured afferents is reinforced by channel remodeling [37]. Pain signaling in Aδ and C fibers depends on voltage-gated sodium channels (NaV) that generate and propagate action potentials. By adjusting peripheral NaV activity, nociceptive drive can be reduced before central amplification takes place [38]. Thermo-transient receptor potential channels (thermo-TRPs) on sensory nerve endings connect signals from inflammation with temperature changes. Substances like prostanoids and cytokines make these channels more sensitive, linking tissue inflammation with stronger pain responses [39,40]. Therefore, a logical way to prevent early amplification of neuropathic pain is to strategically target peripheral channel function and inflammatory mediators.

Peripheral events propagate centrally through both neural and vascular routes. Although the spinal cord is protected by the blood-medullary (blood–spinal cord) barrier, peripheral nerve injury can disrupt its integrity, permitting influx of circulating immune cells into the dorsal horn milieu [37]. At the same time, glial cells change their behavior [41]. Microglia multiply, become motile and phagocytic, show new receptors, and release pro-inflammatory signals [41,42]. Astrocytes also shift into a reactive state, keeping up the production of cytokines and chemokines [43]. Coordinated glial cell recruitment and activation cause a temporally excessive release of cytokines and chemokines, which fuel neuroinflammation and central sensitization and sustain neuropathic pain states [44]. The precise upstream signals that enforce prolonged glial overproduction after peripheral injury remain incompletely defined and are an active area of investigation [45].

Clinically, these processes result in a diverse set of symptoms that combine “positive” characteristics like spontaneous pain, paresthesia, and dysesthesia with “negative” indicators like sensory loss and, occasionally, cognitive or motor impairment [46]. From a mechanistic perspective, this represents long-lasting modifications in ion-channel expression, synaptic strength, inhibitory–excitatory balance, and gene transcription along pain pathways [1]. Understanding how cytokine signaling, peripheral neuroinflammation, ion-channel plasticity, barrier dysfunction, and glial activation contribute in stages aids in early diagnosis and mechanism-targeted treatments meant to break the vicious cycle of inflammation and excitation.

## 3. Inflammation and Neuropathic Pain

Neuropathic pain unfolds within a sustained inflammatory context that spans the injured peripheral nerve, dorsal root ganglia (DRG), and spinal cord [47,48]. Following nerve damage, resident Schwann cells and macrophages rapidly release danger-associated molecular patterns (DAMPs) and eicosanoids, while endothelial activation increases leukocyte adhesion and extravasation [27]. Monocytes, macrophages, lymphocytes, and plasma cells accumulate at the lesion, where they generate reactive oxygen and nitrogen species (ROS/RNS) and secrete cytokines and chemokines that intensify local excitability [27,49]. This influx of cells is not just a side effect. It changes the properties of pain-sensing nerves, making them more excitable, lowering the threshold needed to activate them, and causing abnormal ongoing firing. As a result, these nerves keep sending signals that continuously stimulate central pain circuits [50].

Pattern-recognition receptors (PRRs), especially Toll-like receptor 4 (TLR4) found on macrophages, microglia, and even pain-sensing neurons, detect danger signals. When activated, they trigger intracellular signaling through the myeloid differentiation primary response 88 (MyD88) and TIR-domain-containing adapter-inducing interferon-β (TRIF) pathways [51]. These signaling routes converge at NF-κB and MAPKs, including p38, c-Jun N-terminal kinase (JNK), and extracellular signal-regulated kinase (ERK), which switch on genes that produce TNF, IL-1β, IL-6, COX-2, and chemokines such as C-C motif chemokine ligand 2 (CCL2) and C-X3-C motif chemokine ligand 1 (CX3CL1) [52,53]. At the same time, interleukin-6 family cytokines activate the glycoprotein 130 (gp130)/Janus kinase–signal transducer and activator of transcription (JAK–STAT) pathway, which helps maintain inflammation and promotes astrocyte reactivity [53,54]. Prostaglandin E_2_, produced by COX-2, acts on EP receptors located on primary sensory neurons and dorsal horn neurons. This interaction enhances cyclic adenosine monophosphate–protein kinase A/protein kinase C (cAMP–PKA/PKC) signaling, promotes phosphorylation of ion channels, and strengthens synaptic transmission. In this way, cytokine production is directly linked to increased pain sensitivity [53,54].

Peripheral inflammation directly modulates nociceptor transduction [55]. Tumor necrosis factor and IL-1β increase Nav channel availability and suppress repolarizing potassium (K^+^) currents, thereby favoring repetitive neuronal firing [56,57]. Prostaglandin E_2_, oxidized lipids, and electrophiles sensitize transient receptor potential vanilloid 1 (TRPV1) and activate transient receptor potential ankyrin 1 (TRPA1) through covalent or redox interactions, thereby linking lipid peroxidation to thermal and chemical hyperalgesia [58]. Chemokine axes, such as C-C motif chemokine ligand 2–C-C chemokine receptor type 2 (CCL2–CCR2) and C-X3-C motif chemokine ligand 1–C-X3-C chemokine receptor 1 (CX3CL1–CX3CR1), recruit and position immune cells but also signal within neurons to enhance excitability [59,60]. Sensory neurons reciprocate by releasing pro-inflammatory mediators such as adenosine triphosphate (ATP), substance P, calcitonin gene-related peptide (CGRP), and various cytokines, which amplify vascular permeability and immune cell influx—thereby forming a bidirectional neuron–immune loop that resists spontaneous resolution [55].

Central neuroinflammation consolidates chronicity [61]. The movement of immune cells and DAMPs into the dorsal horn is made possible by peripheral input and barrier disruption at the blood–nerve and blood–spinal cord interfaces [62]. Microglia adopt a reactive phenotype characterized by NADPH oxidase 2 (NOX2)-driven ROS production and p38 MAPK activation, releasing IL-1β, TNF, and brain-derived neurotrophic factor (BDNF) [63,64]. Brain-derived neurotrophic factor activates its receptor tropomyosin receptor kinase B (TrkB), leading to downregulation of potassium-chloride cotransporter 2 (KCC2) in dorsal horn neurons. This process diminishes the inhibitory effects normally mediated by gamma-aminobutyric acid (GABA) and glycine, resulting in disinhibition—a defining feature of central sensitization [65]. Astrocytes sustain the inflammatory response through STAT3 and NF-κB signaling. These pathways maintain COX-2 activity, disrupt the physiological balance of glutamate, and promote the release of chemokines [66]. As a consequence, microglia remain activated and immune cells persist within the tissue. Together, these glial changes turn short-term pain signals into long-lasting strengthening of synapses and increased network excitability.

Oxidative stress interlocks with inflammation at every tier. Cytokine and TLR signaling activate NOX enzymes and inducible nitric oxide synthase (iNOS), which increase the production of ROS and RNS [67]. These molecules further stimulate NF-κB and MAPK pathways, while also inflicting damage on mitochondrial and nuclear DNA [68]. Mitochondrial danger signals and oxidized lipids activate the cyclic GMP–AMP synthase–stimulator of interferon genes (cGAS–STING) pathway and the NOD-, LRR-, and pyrin domain-containing protein 3 (NLRP3) inflammasome. This activation promotes the maturation of IL-1β and further amplifies the release of pro-inflammatory cytokines [69]. This cycle of oxidative stress and inflammation explains what is often seen in practice. Stronger inflammation is linked to stronger pain. By calming either the inflammatory or oxidative stress pathways, pain sensitivity can be reduced, even if the original injury itself does not change.

Neuropathic pain develops when several processes work together: immune cells move into the area, receptors sense danger signals and trigger cytokine release, prostanoids are produced, and glial cells remain active. Together, these create an inflammatory environment that keeps lowering the threshold for neurons to fire. To break this cycle, treatments need to match the mechanisms involved. Examples include blocking TLR4/NF-κB or JAK–STAT signaling, inhibiting COX-2 or EP receptors, and targeting chemokine systems like CCL2/CCR2 or CX3CL1/CX3CR1. Adding therapies that control oxidative stress, such as NOX or iNOS modulation, and treatments aimed at glial cells can strengthen these effects. The goal of these combined approaches is to dismantle the inflammatory support that maintains central sensitization and drives ongoing neuropathic pain.

*Peripheral and Central Inflammation and the Role of Glial Cells in Neuropathic Pain*

**Figure 2:**
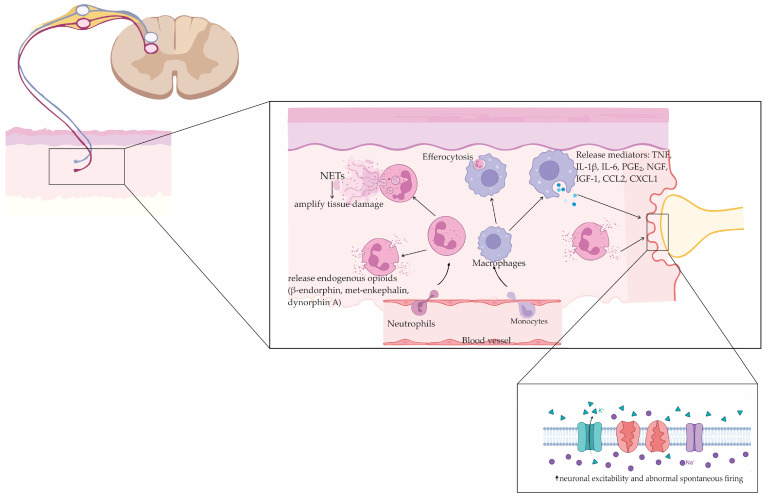
Peripheral immune orchestration of nociceptor sensitization at an injured nerve. Overview of the peripheral tissue environment adjacent to a nociceptor terminal. Neutrophils provide early host defense and opioid-mediated analgesia but, under persistent inflammation, form NETs that aggravate tissue injury. Efferocytosis by macrophages clears apoptotic neutrophils and supports resolution. Monocytes entering from the vasculature differentiate into macrophages that produce TNF, IL-1β, IL-6, PGE_2_, NGF, IGF-1, CCL2, and CXCL1 in the perineuronal space. These mediators act on the nociceptor ending to increase TRPA1/TRPV1 activity and the availability of Nav1.7–Nav1.9 channels, culminating in increased neuronal excitability and spontaneous activity.

Peripheral inflammation sets the stage for chronic pain by recruiting and reprogramming innate and adaptive immune cells at and around the injured nerve [9,70]. As shown in Figure 2, neutrophils arrive first and can be analgesic in the acute phase by releasing endogenous opioids (β-endorphin, met-enkephalin, dynorphin A) and clearing pathogens [32]. When inflammation persists, they form neutrophil extracellular traps and amplify tissue damage [32]. Efficient efferocytosis—macrophage uptake of apoptotic neutrophils—helps terminate this response and accelerates pain resolution [71]. Monocytes differentiate into macrophages that orchestrate phagocytosis, antigen presentation, and cytokine production [72]. In the area around pain-sensing neurons, macrophages release molecules such as TNF, IL-1β, IL-6, PGE_2_, nerve growth factor (NGF), insulin-like growth factor 1 (IGF-1), and chemokines including CCL2 and C-X-C motif chemokine ligand 1 (CXCL1). These substances directly make the primary sensory neurons more sensitive by changing how ion channels, for example, by altering TRPA1 and TRPV1 activity, or by increasing the availability of voltage-gated sodium channels Nav1.7–Nav1.9 [73,74]. As a result, neurons become more excitable and may exhibit abnormal firing. Neuron–macrophage crosstalk is reinforced by CCL2/CCR2 signaling between DRG neurons and infiltrating macrophages [75]. In parallel, DAMPs such as high-mobility group box 1 (HMGB1) engage TLR4 and the receptor for advanced glycation end products (RAGE), and accelerate a CXCL12/CXCR4 axis within peripheral nerves, deepening nociceptor sensitization [73,76,77].

Macrophages exhibit functional plasticity that shapes pain trajectories [72]. Pro-inflammatory “M1-like” macrophages, triggered by signals such as lipopolysaccharide (LPS), granulocyte-macrophage colony-stimulating factor (GM-CSF), interferon-gamma (IFN-γ), and TNF, increase neuronal excitability and exacerbate tissue damage. In contrast, “M2-like” macrophages, activated by IL-4, IL-10, IL-13, transforming growth factor-beta (TGF-β), and macrophage colony-stimulating factor (M-CSF), contribute to debris clearance, secrete anti-inflammatory mediators, and promote tissue repair [72,78]. Specialized pro-resolving mediators facilitate the transition of macrophages toward a pro-healing phenotype [79]. For instance, neuroprotectin D1 (NPD1) acts on the G protein-coupled receptor 37 (GPR37) in macrophages, enhancing their capacity for debris clearance and increasing IL-10 production. This action shortens the duration of inflammation and contributes to pain resolution [80]. Thus, inflammation can be both foe (sensitization) and friend (resolution), depending on cellular programming and timing [32].

Central inflammation consolidates chronicity through glial mechanisms that convert transient peripheral input into durable changes in dorsal horn circuitry [81]. Microglia are the first responders in the spinal cord: within days of nerve injury they proliferate, change morphology, and adopt reactive transcriptional states (“microgliosis”) [42]. Signals from sensory neurons, such as IL-1β, caspase-6, colony-stimulating factor 1 (CSF1), and extracellular proteases, activate microglia. Once activated, microglia release pro-inflammatory and modulatory molecules, including TNF, IL-1β, PGE_2_, and BDNF. These factors further amplify pain signaling and contribute to central sensitization [42,72]. Brain-derived neurotrophic factor activates its receptor TrkB on dorsal horn neurons, leading to reduced activity of KCC2. Impaired KCC2 function disrupts chloride homeostasis, thereby weakening inhibition mediated by GABA and glycine. This loss of inhibition, termed disinhibition, represents a hallmark feature of central sensitization [65]. Microglia express a variety of receptors and ion channels that render them highly responsive to environmental signals. Adenosine triphosphate-gated receptors P2X purinoceptor 4 (P2X4) and P2X purinoceptor 7 (P2X7) activate microglia to release cytokines and initiate assembly of NLRP3 inflammasome through caspase-1 [82,83]. The voltage-gated proton channel H_v1 facilitates the generation of ROS via NOX enzymes and supports communication between microglia and astrocytes [84]. Following peripheral nerve injury, transient receptor potential vanilloid 4 (TRPV4) is upregulated, resulting in the release of lipocalin-2 (LCN2), which contributes to enhanced pain sensitivity [85]. Notably, microglial activation is most prominent early and tends to wane after weeks, which helps explain why microglia-targeted inhibitors show greatest efficacy as preventive or early interventions [63,86]. In line with current consensus, the M1/M2 nomenclature is considered obsolete; instead, microglia are described using context-specific, transcriptionally defined states by transcriptional programs and function [87]. Single-cell studies reveal discrete non-homeostatic states—such as disease-associated microglia (DAM) and injury/activated-response states—that emerge in neurodegeneration and after nerve injury [87].

After peripheral nerve injury, microglia engage a neuron–microglia communication pathway centered on fractalkine (CX3CL1) and its microglial receptor CX3CR1, which has emerged as a consistent feature across neuropathic pain models [88]. In the dorsal horn, membrane-anchored neuronal CX3CL1 is proteolytically shed by microglial cathepsin-S to generate soluble CX3CL1 that activates CX3CR1 and propagates pain signaling [89]. Blocking spinal cathepsin-S reverses established mechanical hypersensitivity in nerve-injured rats, functionally linking Cat-S–driven CX3CL1 release to maintenance of neuropathic pain [90].

In experimental autoimmune neuritis—the rat model of Guillain–Barré syndrome—mechanical allodynia appears days before neurological deficits and coincides with increased dorsal-horn microglia, indicating early central sensitization [91]. Within this model, spinal CX3CL1/CX3CR1 expression is upregulated, supporting a role for fractalkine signaling in pain associated with peripheral inflammatory neuropathies [91].

Astrocytes, more abundant than microglia, enter a reactive state (“astrogliosis”) slightly later but persist for months, thereby maintaining central sensitization [92]. When astrocytes adopt a reactive phenotype, they upregulate glial fibrillary acidic protein (GFAP) expression and begin to proliferate. Rather than carrying out their normal supportive functions—such as maintaining K^+^ homeostasis, clearing glutamate, and regulating water balance—they shift toward the production of pro-inflammatory mediators [32]. In this state, astrocytes continuously express COX-2 and PGE_2_, release cytokines and chemokines including CCL2, CXCL1, and CX3CL1, and generate NO via iNOS. Collectively, these changes sustain inflammation and amplify pain signaling. These signals potentiate excitatory transmission, impair inhibitory tone, and stabilize microglial activation, creating a self-reinforcing inflammatory network [32].

**Figure 3:**
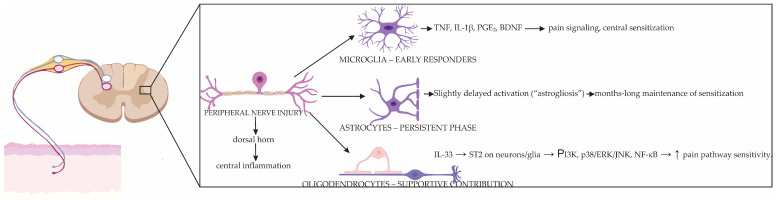
Central glial mechanisms sustaining neuropathic pain. Peripheral nerve injury triggers central inflammation within the dorsal horn of the spinal cord. Microglia are the earliest responders, releasing TNF, IL-1β, PGE_2_, and BDNF that enhance pain signaling and initiate central sensitization. Astrocytes become reactive slightly later (“astrogliosis”) and maintain sensitization for months through persistent cytokine and prostaglandin production. Oligodendrocytes provide a supportive contribution by releasing IL-33, which activates ST2 receptors on neurons and glia to engage PI3K and MAPK (p38/ERK/JNK) and NF-κB signaling, thereby increasing pain pathway sensitivity. Together, these glial interactions create a self-reinforcing inflammatory network that stabilizes central sensitization and chronic pain.

Oligodendrocytes, although less frequently studied in the context of pain, also contribute to its modulation [93]. Following injury in the dorsal spinal cord, they release interleukin-33 (IL-33). This cytokine binds to its receptor suppression of tumorigenicity 2 (ST2) expressed on neurons and glial cells, thereby activating phosphoinositide 3-kinase (PI3K) and MAPK pathways, including p38, ERK, JNK, as well as nuclear factor NF-κB signaling. These cascades enhance the sensitivity of pain pathways and promote nociceptive transmission [94]. Interactions among oligodendrocytes, microglia, astrocytes, and primary afferents likely fine-tune conduction and synaptic integration in ways that remain under active investigation. Figure 3 summarizes the key cellular interactions that sustain this process.

In sum, Neuropathic pain develops when immune, neuronal, and glial mechanisms reinforce one another to maintain a chronic inflammatory state. At the periphery, immune cell recruitment and macrophage signaling sensitize nociceptors, lowering their activation threshold and causing abnormal firing. In the spinal cord, microglia respond early to neuronal cues through receptors such as P2X4, P2X7, TLR4, and CSF1, releasing pro-inflammatory factors that embed these signals into neural circuits. Over time, astrocytes take on the role of sustaining inflammation through COX-2 activity, prostaglandin release, chemokine output, and glutamate imbalance, while even oligodendrocytes contribute by releasing IL-33 that further activates pain pathways. Because these processes evolve with time, therapeutic timing is crucial: early interventions may be most effective when targeting microglial receptors or upstream triggers, whereas persistent pain may respond better to astrocyte-directed strategies such as COX-2 or EP receptor inhibition and chemokine blockade. Combining these with approaches that promote resolution—such as specialized pro-resolving mediators, macrophage reprogramming toward an M2 phenotype, and enhanced efferocytosis—offers a way to dismantle the inflammatory support system that stabilizes central sensitization and sustains neuropathic pain. To integrate these findings, Table 1 maps peripheral and central inflammatory cascades to their effects on nociceptive processing and to actionable clinical strategies.

## 4. Oxidative Stress in Neuropathic Pain: Sources, Redox Signaling, and Effects on Nociceptive Circuits

**Figure 4:**
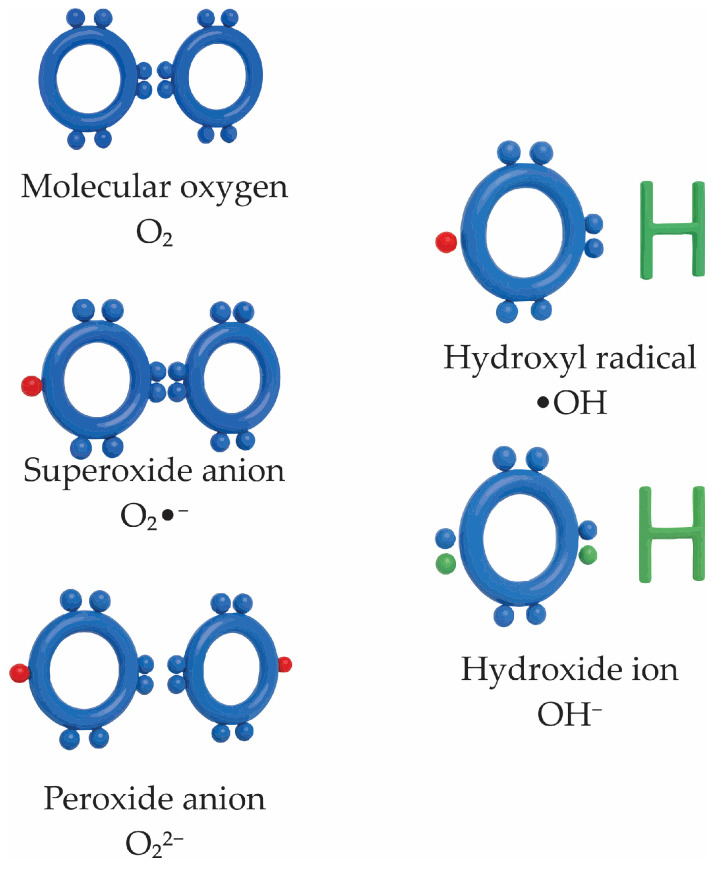
Representative reactive oxygen species relevant to oxidative stress. Schematic depiction of major ROS, including molecular oxygen (O_2_), superoxide anion (O_2_•^−^), peroxide anion (O_2_^2−^), hydroxyl radical (•OH), and hydroxide ion (OH^−^). Although these species can mediate physiological redox signaling at low levels, their accumulation drives oxidative damage to nucleic acids, proteins, and lipids, contributing to cellular dysfunction and disease progression.

Oxidative stress emerges from a pathological shift in the cellular redox balance, where ROS and RNS exceed the buffering capacity of antioxidant systems [95,96]. This imbalance disrupts redox signaling and promotes damage to DNA, proteins, and lipids, ultimately contributing to cellular dysfunction and degeneration [97]. Central to this phenomenon is the overaccumulation of ROS, such as superoxide anion (O_2_•^−^), hydrogen peroxide (H_2_O_2_), and hydroxyl radical (•OH) (Figure 4), alongside RNS species including nitric oxide (NO) and peroxynitrite (ONOO^−^) [98]. Although these species can participate in physiological signaling at low concentrations, their excess triggers a cascade of deleterious biochemical reactions that impair cellular integrity and survival [97].

In neurons, mitochondria represent the principal endogenous source of ROS [99]. Within the mitochondrial electron transport chain (ETC), complexes I and III leak electrons that prematurely reduce molecular oxygen, generating superoxide [100]. This process is exacerbated under metabolic stress, aging, or impaired glucose utilization—conditions known to compromise mitochondrial efficiency and elevate radical production [101,102]. Additionally, peroxisomes, endoplasmic reticulum, and enzymatic systems such as NADPH oxidase isoforms (NOX1–5), dual oxidases (Duox1/2), and various nitric oxide synthases (NOS1–3) further contribute to intracellular ROS and RNS flux [103,104]. Notably, NOX enzymes are dedicated sources of regulated ROS for immune and signaling functions, yet their hyperactivation results in oxidative injury, especially within the nervous system [105,106,107].

Exogenous factors also intensify oxidative load, especially in environmentally exposed tissues. Ultraviolet (UV) radiation promotes the formation of mutagenic DNA lesions like 8-oxo-guanine and depletes key antioxidants such as glutathione (GSH) [108,109]. Ionizing radiation increases intracellular peroxide accumulation, while alpha particles enhance oxygen consumption and accelerate ROS generation in fibroblasts [110,111]. Toxic metals such as iron, copper, cadmium, and arsenic can accelerate chemical reactions that generate highly reactive molecules. In particular, they drive the Fenton and Haber–Weiss reactions, which produce hydroxyl radicals. These radicals are extremely damaging, as they attack and oxidize DNA, RNA, and proteins, leading to structural injury and loss of function in cells [107]. Exposure to ozone leads to airway inflammation and oxidant-mediated respiratory dysfunction, even in healthy individuals. Furthermore, xenobiotics such as pesticides, cigarette smoke, and industrial pollutants serve as potent ROS inducers through diverse mechanisms of redox cycling and mitochondrial interference [107].

Beyond cellular damage, redox imbalance critically modulates intracellular signaling cascades. One key redox-sensitive transcription factor is NF-κB, whose activation governs pro-inflammatory gene expression. ROS can modify cysteine residues on NF-κB or its upstream kinases, altering DNA binding capacity and transcriptional activity [95,112]. However, cellular redox regulators such as thioredoxin (Trx) and redox factor-1 (Ref-1) may restore NF-κB function by reducing oxidized cysteines [113,114]. Electrophilic lipid mediators, including cyclopentenone prostaglandins, antagonize ROS-mediated inflammation by covalently modifying redox-sensitive proteins, thereby mitigating NF-κB activation [115].

Importantly, sustained oxidative stress induces genomic instability, particularly double-strand DNA breaks, which are frequently observed in cancer and neurodegeneration [116,117]. These lesions may arise from non-enzymatic mitochondrial processes or from aberrant enzymatic activity under inflammatory conditions [96]. Thus, oxidative stress is not merely a byproduct of cellular dysfunction but a driver of pathophysiology, especially in tissues with high metabolic demands such as the central nervous system.

### 4.1. Oxidative Stress and Neuropathic Pain

Oxidative stress is a core driver of neuropathic pain, operating both as a consequence of nerve injury and as an amplifier of neuroinflammation and neuronal hyperexcitability [99]. The nervous system is intrinsically vulnerable because of high oxygen use, abundant polyunsaturated lipids, and long, mitochondria-rich axons [118,119]. After a peripheral or central injury, the normal balance between oxidants and antioxidants is disrupted. Instead of being tightly controlled, ROS and RNS stay elevated for long periods. Signals that would normally help the cell adapt are turned into harmful processes, causing oxidation and nitration of proteins, lipids, and nucleic acids throughout the tissue [104]. This redox imbalance couples to innate immune pathways, sustains cytokine output, and lowers activation thresholds across pain circuits [95,120].

Reactive oxygen and nitrogen species in the body primarily originate from specific enzymes and organelles [104]. Mitochondria represent a major source: when electrons leak from complex I or from the ubiquinol oxidation (Q_o) site of complex III, molecular oxygen (O_2_) is reduced to superoxide (O_2_•^−^) [121]. This unstable molecule is rapidly converted into hydrogen peroxide (H_2_O_2_) by mitochondrial superoxide dismutase 2 (SOD2) [122]. In the presence of free ferrous iron (Fe^2+^) or cuprous copper (Cu^+^) ions, H_2_O_2_ undergoes the Fenton reaction, generating hydroxyl radicals (•OH) [123]. These radicals are highly reactive and capable of inflicting severe cellular and molecular damage. Nicotinamide adenine dinucleotide phosphate oxidases—including NOX1, NOX2, NOX4, and dual oxidases 1 and 2 (DUOX1/2)—function as stimulus-coupled generators of superoxide (O_2_•^−^) and hydrogen peroxide (H_2_O_2_) in neurons, Schwann cells, microglia, and endothelial cells [124,125,126]. Nitric oxide synthases (nNOS, iNOS, eNOS) produce nitric oxide (NO•), which reacts with O_2_•^−^ to yield peroxynitrite (ONOO^−^), a potent oxidant capable of inactivating mitochondrial enzymes such as aconitase and complex I, as well as damaging nuclear and mitochondrial DNA [127,128,129]. Additional sources of oxidative stress include endoplasmic reticulum (ER) oxidative protein folding mediated by endoplasmic reticulum oxidoreductin 1–protein disulfide isomerase (ERO1–PDI) and peroxisomal β-oxidation, both of which contribute to elevated H_2_O_2_ levels [130]. Pathological conditions such as tissue injury, hyperglycemia, chemotherapeutic exposure, aging, and defective mitophagy further exacerbate these fluxes, while insufficient nuclear factor erythroid 2–related factor 2 (Nrf2) activity or depletion of GSH reduces antioxidant buffering capacity—ultimately locking tissues into a chronic pro-oxidant state [97,131].

At the nociceptor, ROS or RNS directly remodel excitability [132]. Electrophiles and lipid peroxidation products, such as 4-hydroxynonenal (4-HNE), isoprostanes, and oxidized phospholipids, covalently modify TRPA1 and sensitize TRPV1, thereby lowering thermal and chemical activation thresholds and increasing channel open probability [133,134]. Nerve growth factor- driven lipid oxidation further potentiates TRPV1 function [135]. Redox modifications significantly influence voltage-gated ion channel activity. Nitration or S-nitrosylation of NaV channel subunits facilitates repetitive neuronal firing, while oxidation of voltage-gated potassium (Kv) channels diminishes their repolarizing capacity. In addition, hyperpolarization-activated cyclic nucleotide-gated (HCN) channel gating can shift to favor ectopic activity [132,136]. Mitochondrial dysfunction within axons impairs ATP-dependent pumps and Ca^2+^ homeostasis, promoting spontaneous discharges [137]. Collectively, these peripheral effects magnify the afferent barrage entering the spinal cord.

In the dorsal horn, redox signaling stabilizes central sensitization [107]. Microglia transition to a pro-inflammatory state with NOX2- and mitochondria-derived ROS, activating p38/JNK and NF-κB pathways to release IL-1β, TNF, and BDNF [63,64]. BDNF–TrkB signaling downregulates the K^+^–Cl^−^ cotransporter KCC2, weakening GABA/glycine-mediated inhibition and producing disinhibition [65]. Astrocytes, via STAT3 and NF-κB, sustain COX-2–PGE_2_ production and glutamate–glutamine cycle dysregulation; PGE_2_ acting at EP receptors enhances synaptic gain [138]. Redox changes can directly modulate neuronal mechanisms that amplify pain signaling. Specific residues on N-methyl-D-aspartate (NMDA) receptor subunits, including glutamate ionotropic receptor NMDA type subunit 1 (GluN1) and subunit 2 (GluN2), are highly sensitive to oxidative modifications, which enhance receptor activity [139]. Moreover, S-nitrosylation—a process in which NO covalently attaches to cysteine residues on synaptic proteins—further augments excitatory signaling [140]. Collectively, these modifications facilitate long-term potentiation within pain pathways, reinforcing and amplifying nociceptive input [139,140]. Peroxynitrite adds nitrate groups to proteins inside both mitochondria and the cytoplasm of dorsal horn neurons and glial cells. This damages enzymes needed for energy production and weakens antioxidant defenses. As a result, the cells lose stability, and their excitability remains high, which helps keep pain pathways overactive [141].

Oxidants also act as immune amplifiers. Mitochondrial DNA (mtDNA) leakage and oxidized DAMPs activate cGAS–STING pathway as well as TLR and RAGE signaling [142]. These cascades enhance NF-κB and interferon regulatory factor (IRF) transcriptional programs, while also promoting NLRP3 inflammasome activation, which drives the maturation of IL-1β and propagates neuroinflammation [143]. This biochemical–immune coupling links peripheral injury to sustained central cytokine excess.

### 4.2. Mitochondrial Dysfunction in Neuropathic Pain

Neurons rely on mitochondria to meet the high energetic cost of excitability and synaptic transmission [144]. ATP generated by oxidative phosphorylation powers Na^+^/K^+^-ATPase to re-establish membrane potentials after spikes and fuels presynaptic vesicle cycling and axonal transport [145]. Beyond bioenergetics, mitochondria shape intracellular Ca^2+^ dynamics. They buffer activity-dependent Ca^2+^ elevations through the mitochondrial calcium uniporter (MCU) and release Ca^2+^ via the mitochondrial sodium/calcium exchanger (NCLX), thereby modulating neurotransmitter release probability and shaping short-term synaptic plasticity [137,146]. In sensory neurons with long axons, the correct positioning of mitochondria at nodes of Ranvier and terminals is essential; impaired transport or local failure lowers energy reserve and predisposes to ectopic firing [147].

Mitochondrial quality control is preserved through a tightly regulated cycle of fusion and fission, coupled with organelle turnover. Fusion is mediated by mitofusin-1/2 (MFN1/2) and optic atrophy protein 1 (OPA1), whereas fission is driven by dynamin-related protein 1 (DRP1). These dynamics are coordinated with biogenesis, regulated by peroxisome proliferator-activated receptor gamma coactivator 1-alpha (PGC-1α), nuclear respiratory factors 1 and 2 (NRF1/2), and mitochondrial transcription factor A (TFAM). Damaged mitochondria are selectively removed through mitophagy, a process orchestrated by PTEN-induced kinase 1 (PINK1) and the E3 ubiquitin ligase Parkin [148]. Disruption at any point in this network—such as DRP1-driven mitochondrial fragmentation, mitofusin-2 (MFN2) dysfunction, impaired biogenesis, or defective mitophagy—leads to the accumulation of depolarized, ROS-rich mitochondria within axons and somata [149]. Membrane contact sites with ER, termed mitochondria-associated membranes (MAMs), coordinate lipid exchange and inositol 1,4,5-trisphosphate receptor (IP_3_R)-mediated Ca^2+^ transfer. Under pathological conditions, maladaptive ER–mitochondria coupling promotes Ca^2+^ overload, cyclophilin D-dependent opening of the mitochondrial permeability transition pore (mPTP), and ultimately bioenergetic collapse [149,150].

Mitochondria are the dominant endogenous source of reactive oxygen species in neurons [100]. Electron leak at complex I and at the Qo site of complex III reduces O_2_ to superoxide, which SOD2 converts to H_2_O_2_; in the presence of labile iron or copper, H_2_O_2_ yields hydroxyl radicals via Fenton chemistry [123]. When cardiolipin, a key lipid in the inner mitochondrial membrane, becomes oxidized, it disrupts the stability of respiratory supercomplexes that normally organize the electron transport chain. This destabilization weakens efficient energy transfer and loosens cytochrome c from the cristae structure. The freed cytochrome c both amplifies the production of reactive oxygen and nitrogen species and promotes swelling of mitochondria. Ultimately, it leaks into the cytosol, where it signals cell stress or death, while the mitochondria themselves lose their ability to carry out normal oxidative phosphorylation and energy production [151,152]. When antioxidant systems (e.g., glutathione, peroxiredoxins, thioredoxin) are depleted or outpaced, ROS or RNS oxidize proteins, lipids, and DNA, initiating DNA-damage responses and long-lived changes in neuronal function [149].

These mitochondrial derangements couple directly to the initiation and persistence of neuropathic pain [148]. When energy production drops, the Na^+^/K^+^-ATPase pump works less effectively. This makes it harder for neurons to maintain their resting potential, so Aδ and C fibers become unstable and start firing on their own [132]. At the same time, calcium balance is disturbed, leading to excess neurotransmitter release and activation of calcium-dependent kinases. These kinases add phosphate groups to ion channels, which makes neurons even more excitable [132]. Reactive oxygen and nitrogen species and lipid peroxidation products—such as 4-HNE and isoprostanes—covalently modify TRPA1and sensitize TRPV1, thereby lowering thermal and chemical activation thresholds. In parallel, redox modifications of NaV channels and Kv channels facilitate high-frequency neuronal firing and diminish the repolarizing reserve [153]. Mitochondrial NO• and peroxynitrite nitration deactivate key metabolic enzymes (e.g., aconitase, complex I), deepening energy failure and increasing oxidative load—a self-reinforcing loop that sustains peripheral hyperexcitability [148,153].

Structural and functional evidence supports this link across pain models. Ultrastructural studies show clear mitochondrial damage in different types of nerve cells. In peripheral sensory axons, DRG neurons, and Schwann cells, mitochondria appear swollen and full of vacuoles. This pattern is seen in conditions such as diabetes, chemotherapy-induced neuropathy, and experimental models like sciatic nerve constriction. These changes are consistent with mitochondria losing their ability to breathe properly and regulate calcium, which are both essential for healthy nerve function [154]. Manipulating mitochondrial function in nociceptors modifies pain phenotypes: pharmacologic or genetic interventions that restore respiration, limit ROS, or normalize Ca^2+^ flux reduce mechanical and thermal hypersensitivity in neuropathic and inflammatory models [154,155]. Hyperalgesic priming offers a mechanistic example of how mitochondrial dysfunction sustains pain. Transient inflammation elevates ATP synthase c-subunit lysine N-methyltransferase (ATPSc-KMT), which alters respiratory efficiency and impairs the resolution of pain. Experimental interventions—such as knocking down ATPSc-KMT, moderating mitochondrial respiration, or supplementing depleted metabolites—restore resolution and prevent the transition to chronic pain [156].

Therapeutically, mitochondria offer multiple actionable targets. Upstream strategies reduce oxidant production (e.g., limiting reverse electron transport at complex I, optimizing substrate supply, or recoupling NOS), while downstream approaches stabilize the organelle (cardiolipin-targeted peptides), enhance quality control (PGC-1α agonism, mitophagy support), or directly scavenge mitochondrial ROS (matrix-targeted antioxidants). Because energetic, Ca^2+^, and redox axes are interdependent, combination regimens—pairing mitochondrial support with glial modulators or redox-gated channel antagonists—are most likely to disrupt the bioenergetic–inflammatory–excitatory loop that maintains neuropathic pain. A concise mapping of oxidative nodes to pain mechanisms and actionable therapies is provided in Table 2.

## 5. Cellular Senescence in Neuropathic Pain: Triggers, SASP, and Neural Consequences

Cellular senescence, first described by Hayflick and Moorhead in the early 1960s, refers to the irreversible cessation of cell proliferation after a finite number of divisions [96]. Initially characterized in human fibroblasts, this process—termed replicative senescence—was later attributed to progressive telomere shortening, which induces chromosomal instability and functions as a tumor-suppressive mechanism [157,158]. Over time, senescence has been recognized as a multifaceted biological program that operates not only as a safeguard against malignant transformation but also as a participant in diverse physiological processes, including embryonic development, wound healing, and tissue regeneration [159,160]. Senescent cells remain metabolically active and adopt a distinctive secretory profile known as the senescence-associated secretory phenotype (SASP), which comprises pro-inflammatory cytokines, chemokines, growth factors, and proteases [161]. While SASP can facilitate tissue remodeling and repair, its chronic activation drives persistent low-grade inflammation, thereby contributing to aging and the pathogenesis of age-related diseases [162].

Different forms of cellular senescence, such as replicative, oncogene-induced, therapy-induced, stress-related, mitochondrial dysfunction–associated, and immune-driven, share common traits [163]. They all involve the activation of cell cycle inhibitors, including p53, p21^WAF1/Cip1^, and p16^INK4a^, as well as the activity of senescence-associated β-galactosidase (SA-β-gal) [164]. However, the exact molecular features and the functional consequences vary depending on the type of stress that triggered the process and the specific cellular environment.

### 5.1. Sources and Types of Cellular Senescence

**Figure 5:**
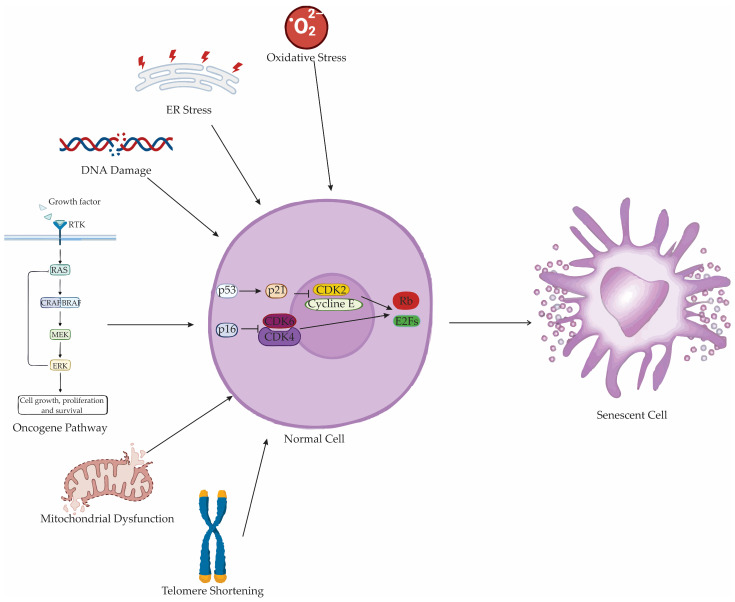
Major triggers and checkpoint pathways driving cellular senescence. Schematic overview of key senescence inducers—oxidative stress, endoplasmic-reticulum (ER) stress, DNA damage/DDR activation, oncogenic signaling, mitochondrial dysfunction, and telomere shortening—converging on canonical checkpoint modules within a normal cell. p53 stabilization with p21 inhibits CDKs; p16^INK4A^ restrains CDK4/6; CDK2–Cyclin E control is depicted together with the Rb–E2F node. The outcome is durable cell-cycle arrest and a senescent phenotype characterized by enlarged morphology and secretory activity.

Senescence can be initiated by a variety of physiological and pathological stressors that converge on pathways enforcing irreversible cell cycle arrest (Figure 5) [164]. Classic triggers include telomere attrition, which underlies replicative senescence, and persistent DNA damage, commonly manifesting as double-strand breaks that activate the DNA damage response (DDR) [165]. The DNA damage response signaling cascade recruits key factors such as phosphorylated histone H2AX (γH2AX), mediator of DNA damage checkpoint protein 1 (MDC1), and p53-binding protein 1 (53BP1) to sites of DNA lesions [166,167,168]. This recruitment sustains activation of the ataxia-telangiectasia mutated (ATM), ataxia-telangiectasia and Rad3-related (ATR), checkpoint kinase 1 (CHK1), and checkpoint kinase 2 (CHK2) pathways, which in turn stabilize tumor protein p53 (p53) and promote p21^WAF1/Cip1^-mediated inhibition of cyclin-dependent kinases (CDKs) [168,169].

Oncogene-induced senescence results from aberrant activation of oncogenes such as *NRAS^G12V^* or *BRAF^V600E^*, enforcing growth arrest through p21 and p16 pathways [161,170]. Therapy-induced senescence occurs in response to genotoxic cancer treatments, including chemotherapy and radiation, which trigger DDR activation [171]. Stress-induced senescence is typically telomere-independent and arises from physical or chemical insults, including oxidative stress and DNA-damaging agents [96]. Mitochondrial dysfunction-associated senescence (MiDAS) develops following mitochondrial damage, producing a secretory phenotype distinct from classical SASP and notably lacking IL-1-driven inflammation [172]. Immunologically induced senescence emerges in the presence of excessive pro-inflammatory cytokines, such as IL-17, and is associated with altered Wingless/Integrated (WNT) signaling and extracellular matrix remodeling [173].

### 5.2. Phenotypic Features of Cellular Senescence and the Senescence-Associated Secretory Phenotype

Cellular senescence represents a complex and multifaceted cellular program characterized primarily by irreversible cell cycle arrest and profound changes in cellular morphology, gene expression, and secretory behavior [164]. The arrest is predominantly mediated by CDKs inhibitors such as p21^Cip1/WAF1^ and p16^INK4a,^ which suppress CDK4/6 activity, enforce retinoblastoma (Rb) hypophosphorylation, and consequently silence E2 promoter binding factor (E2F)-dependent transcription required for G1-S progression [164,174,175]. This sustained proliferative arrest distinguishes senescent cells from quiescent or terminally differentiated cells.

Senescent cells acquire distinct morphological features, including an enlarged and flattened shape, increased granularity, and often nuclear irregularities [176,177]. Biochemically, they accumulate lipofuscin and show enhanced activity of lysosomal β-galactosidase, commonly detected as SA-β-gal [177,178]. However, the specificity of SA-β-gal as a standalone biomarker is limited due to its expression in certain non-senescent cells, such as macrophages [179]. Therefore, contemporary approaches recommend a multi-marker strategy, combining SA-β-gal with cell cycle arrest markers such as p16^INK4a^ and p21^Cip1/WAF1^, indicators of DNA damage including γH2AX, and the absence of proliferation markers such as Ki-67, proliferating cell nuclear antigen (PCNA), or bromodeoxyuridine (BrdU) incorporation [180]. Novel detection approaches have emerged, including mass cytometry (CyTOF)-based panels for simultaneous multiparameter assessment and transcriptomic classifiers such as the SenMayo gene set and SenCID, a machine-learning algorithm trained on large senescence transcriptome datasets. These tools aim to delineate senescent cell populations across tissues and pathophysiological states with higher specificity and resolution [181].

A hallmark of senescent cells is their resistance to apoptosis, coupled with the acquisition of a pro-inflammatory and tissue-remodeling secretory profile known as the senescence-associated secretory phenotype (SASP) [182]. The senescence-associated secretory phenotype encompasses a wide repertoire of factors, including interleukins such as IL-1β, IL-6, and IL-8; chemokines such as CCL2, CCL5, and CXCL1; growth factors including TGF-β, growth differentiation factor 15 (GDF15), and epidermal growth factor (EGF); matrix metalloproteinases such as MMP-1 and MMP-3; as well as bioactive lipids [164]. Additionally, SASP includes extracellular vesicles, cytoplasmic chromatin fragments, and non-coding RNAs, which contribute to intercellular communication and paracrine propagation of senescence [164].

Multiple signaling pathways converge to regulate the SASP, notably NF-κB, p38 MAPK, mechanistic target of rapamycin (mTOR), CCAAT/enhancer-binding protein beta (C/EBPβ), and cGAS–STING axis [164]. These pathways are activated in response to persistent DNA damage and other cellular stressors, driving transcriptional programs that reinforce the inflammatory and pro-fibrotic features of the SASP [164]. While SASP factors can exert beneficial effects in acute contexts, such as wound healing and tumor suppression, their chronic activation is implicated in tissue degeneration, immunosenescence, and age-related pathologies [162].

### 5.3. Oxidative Stress as a Driver and Sustainer of Cellular Senescence

Oxidative stress is a central biological force in the initiation and perpetuation of cellular senescence [96]. Excess ROS disturb redox homeostasis [183]. They damage important molecules, especially DNA and mitochondria, and they activate stress signals that force the cell to stop dividing permanently [184,185]. One of the earliest consequences of ROS overload is the activation of DDR, often via oxidative lesions at telomeric repeats [186]. This triggers p53 stabilization and transcriptional induction of cyclin-dependent kinase inhibitors, notably p21^CIP1^ and p16I^NK4a^, culminating in irreversible growth arrest and the formation of senescence-associated heterochromatic foci (SAHF) [168,187,188,189].

Mitochondrial dysfunction represents both a hallmark and a driver of oxidative stress–induced senescence [96]. Damage to mtDNA, which is not histone-protected and is not effectively repaired, compromises the integrity of the respiratory chain, hinders oxidative phosphorylation, and increases electron leakage from complexes I and III, all of which contribute to the production of additional ROS [149,185,190]. This mitochondrial ROS production feeds back to exacerbate nuclear and organellar damage, creating a self-sustaining oxidative loop that stabilizes the senescent phenotype [191]. Peroxisomal insufficiency further aggravates redox imbalance, as catalase-mediated detoxification of hydrogen peroxide is diminished in senescent cells [192,193].

In response to mitochondrial distress, senescent cells attempt to restore energy homeostasis through a signaling cascade involving ATM, protein kinase B, mechanistic target of rapamycin complex 1 (mTORC1), and PGC-1α, which collectively drive mitochondrial biogenesis [194]. However, in the senescent context, these newly formed mitochondria are often functionally compromised, maintaining high ROS output [191]. A parallel metabolic shift toward aerobic glycolysis (Warburg-like effect) provides ATP independent of the impaired electron transport chain but does not resolve chronic energetic stress [195]. This is reflected in altered nicotinamide adenine dinucleotide (NAD^+^) or reduced NADH ratios, sustained activation of AMP-activated protein kinase (AMPK), and stabilization of p21^Cip1/WAF1^ and p16^INK4a^ messenger RNA (mRNA) transcripts via human antigen R (HuR) inhibition, collectively reinforcing p53–retinoblastoma protein checkpoint activation [196,197].

ROS not only activate DDR but also shape the SASP [96]. Through redox-sensitive signaling pathways such as growth arrest and DNA damage-inducible alpha (GADD45A)–p38 MAPK–growth factor receptor-bound protein 2 (GRB2)–TGF-β, oxidative stress sustains p21^Cip1/WAF1^ expression and promotes pro-inflammatory SASP secretion [96]. This secretome propagates paracrine senescence, maintains chronic inflammation, and contributes to tissue degeneration [198]. Mitochondrial ROS and telomeric ROS affect each other in both directions. ROS from mitochondria speed up telomere shortening, while oxidative damage at telomeres weakens mitochondrial function. This two-way interaction keeps redox imbalance locked into the processes that drive cell senescence [199].

The DDR sits at the intersection of oxidative stress and mitochondrial decline [96]. Persistent double-strand breaks, marked by *γH2AX* and *53BP1* foci, maintain checkpoint signaling and SASP expression [166]. DDR activation feeds forward into mitochondrial dysfunction via GADD45A–p38 MAPK signaling, enhancing ROS output and locking cells into a senescent state [96]. Pharmacological ROS scavenging, particularly when combined with mTOR inhibition, can restore mitochondrial membrane potential, reduce double-strand breaks, and partially reverse senescence-associated defects.

The p53–p21 and p16–Rb pathways are principal executors of oxidative stress–driven growth arrest [96]. Reactive oxygen species -activated ATM and ATR kinases phosphorylate CHK1 and CHK2, leading to p53 stabilization, p21^Cip1/WAF1^ induction, and enforcement of G1/S and G2/M cell cycle checkpoint blockade [166]. In parallel, ROS or p38 MAPK–driven transcriptional programs increase p16^INK4a^ expression, maintaining Rb in its hypophosphorylated, growth-suppressive state [164]. Together, these pathways silence E2F target genes required for S-phase entry and mitotic progression [200].

An important amplifier of this process is thrombospondin-1 (TSP1), a matricellular glycoprotein upregulated in senescent cells by DNA damage, TGF-β1, and oxidative stress [201]. TSP1 engages CD47 to activate Nox1, increasing ROS production and further stabilizing p53–p21 signaling [201]. This TSP1–CD47–Nox1 axis also inhibits nitric oxide signaling, tilting the redox balance toward oxidative dominance, impairing angiogenesis, and promoting extracellular matrix remodeling in aging tissues [202].

Finally, oxidative stress remodels the senescent cell transcriptome by repressing genes involved in DNA replication and mitosis, altering vesicular trafficking and cell adhesion networks, and upregulating anti-apoptotic B-cell lymphoma 2 (BCL-2) family members. These adaptations enable long-term survival of senescent cells despite cumulative cellular and molecular damage [203]. In this way, ROS are not passive byproducts but active architects of the senescent phenotype, driving both intracellular damage programs and extracellular inflammatory signaling.

### 5.4. Senescence and Inflammation

Inflammation and cellular senescence are intricately linked through a self-reinforcing cycle in which inflammatory signaling not only triggers but also sustains and amplifies the senescent phenotype [204]. Chronic exposure to pro-inflammatory cytokines, chemokines, and lipotoxic signals contributes to the induction of senescence in multiple cell types, often in synergy with oxidative stress and DNA damage [204,205]. This relationship is central to age-related dysfunction, cancer biology, and impaired tissue regeneration [204,205,206].

Senescence can be promoted by persistent inflammatory stimuli, including IL-1β, IL-6, IL-8, TNF-α, and C-C motif chemokine ligand 11 (CCL11), which act through redox-sensitive pathways and transcriptional programs such as NF-κB, C/EBPβ, and p38 MAPK [198]. These pathways converge on cell cycle regulators like p16^INK4a^, p21^CIP1^, and p15^INK4b^, enforcing growth arrest [207]. Experimental models have demonstrated that exposure to moderate levels of IL-6 or IL-8 induces senescence in pre-damaged or primed cells, with IL-6 acting predominantly via autocrine loops, while IL-8 functions through CXCR2-mediated paracrine signaling [207,208,209]. Inhibition of either cytokine or their receptors delays senescence, underscoring their essential regulatory roles [207].

This inflammatory network is further amplified by feedback mechanisms [207]. For instance, C/EBPβ upregulates IL-6, which in turn sustains C/EBPβ expression, reinforcing the pro-senescent circuit [210]. Simultaneously, the induction of CXCR2 and its ligands (e.g., IL-8, GROα) contributes to a broad chemokine signaling program that stabilizes the senescent state [207]. This mechanism is evident in both replicative and oncogene-induced senescence and is mirrored by the expression patterns observed in premalignant lesions, fibrotic tissue, and aged organs [207].

Inflammation-induced senescence is not restricted to classical cytokines. Lipid-mediated inflammation, such as that driven by dysfunctional adipocytes, promotes immune senescence through the expansion of pro-inflammatory B and T cell subsets, and is linked to telomere attrition and tissue degeneration in metabolic diseases [198]. Furthermore, persistent low-grade inflammation in tissues such as endothelium and epithelium contributes to senescence by driving mitochondrial dysfunction, telomeric instability, and DNA damage—often through ROS-dependent mechanisms [205].

Senescent cells themselves become potent sources of inflammatory mediators via the SASP, perpetuating inflammation both locally and systemically [211]. This SASP-driven immune modulation not only exacerbates tissue dysfunction but also attracts immune cells [162,212]. Interestingly, in cancer models, this process facilitates the clearance of senescent tumor cells by macrophages, NK cells, and neutrophils, demonstrating a dual role of inflammation in both executing and resolving senescence [213].

### 5.5. Senescence and Neuropathic Pain

Once considered exclusive to dividing cells, senescence hallmarks are now documented in post-mitotic neurons and glia that shape pain processing [214,215]. The presence of senescence-like signatures in human pyramidal and cortical neurons, such as p21^CIP1^/p16^INK4a^ induction, SA-β-gal activity, persistent DNA-damage foci, and a pro-inflammatory secretome, suggests a conserved stress program that prevents maladaptive cell-cycle re-entry and may temporarily prevent neuronal loss [215,216,217]. In neurodegenerative settings, genetic or pharmacologic clearance of senescent neurons and glia improves molecular and behavioral outcomes, establishing senescence as a modifiable disease node rather than a mere epiphenomenon [215]. In pain pathways, peripheral nerve injury increases p53-positive neurons, astrocytes, and microglia in the spinal cord, while age and injury drive a senescent phenotype in primary sensory neurons of lumbar DRG, identified by p21/p16 expression, IL-6 upregulation, and SA-β-gal activity [215,218].

Mechanistically, “post-mitotic cellular senescence” (PoMiCS) in neurons is best viewed as a fail-safe response to stress-driven cell-cycle re-entry [214]. Different stressors relevant to neuropathic pain, such as axotomy, restriction of growth factors, chemotherapy, oxidative stress, or genotoxic stress, can push mature neurons into an abnormal attempt to re-enter the S phase of the cell cycle [219]. Induction of cyclin-dependent kinase inhibitors (p21, p16) arrests this trajectory, averts mitotic catastrophe, and preserves cell number [214,220]. The result is functional: continuous activation of checkpoint and DNA-damage pathways traps neurons in a state with altered metabolism and increased secretion [215]. This state changes their normal excitability and disrupts how they integrate signals at the synapse. Thus, transient neuronal senescence may be adaptive early after injury, whereas persistent senescence becomes a pathogenic driver of chronic pain [215].

Senescent nociceptors act as cytokine hubs. After a peripheral injury, some DRG neurons enter a senescent program and begin producing IL-6 as part of their SASP. This IL-6 does not just mirror the signals from macrophages and Schwann cells but actively adds to them, amplifying the local inflammatory response [215,221]. Interleukin-6 signals through gp130/ JAK–STAT pathways in autocrine and paracrine loops to enhance membrane excitability, modulate ion channel expression, and reinforce pain-related transcriptional programs [222,223]. Notably, TRPV1^+^ nociceptors—a neuronal population central to thermal and chemical nociception—expand with age and following injury, while co-expressing p21^Cip1/WAF1^ and IL-6 [215,224]. This provides a direct cellular conduit linking senescence to hyperalgesia [215]. Across traumatic, diabetic, and chemotherapy-induced models, nociceptors consistently show high enrichment of senescence features, linking their sustained SASP output to the maintenance of hypersensitivity.

Glial senescence consolidates central sensitization [218]. Chronic peripheral injury can induce a senescent phenotype in spinal astrocytes, characterized by SA-β-gal positivity, p21/p16 induction, and γH2AX foci, alongside a SASP rich in cytokines, chemokines, and prostanoids that potentiates dorsal horn transmission [218]. This mirrors astrocytic SASP profiles seen in aging-related brain disorders and helps explain the long tail of central neuroinflammation after peripheral lesions [225]. Microglia, likewise, can adopt senescence-like states that shift from trophic support to cytokine production, amplifying neuronal disinhibition and synaptic gain [218]. After spinal cord injury, most senescence-marker-positive cells around lesion borders are neurons, and senescent cortical neurons possess a SASP capable of inducing paracrine senescence in neighboring cells—evidence that senescence signals can propagate within neural microenvironments [226].

These patterns in time and place help refine treatment strategies. Studies show that senolytics given only in the spinal cord bring relief for a short time. This means that clearing senescent cells in the cord is not enough, because the main sources remain outside the cord in the DRG and injured nerves, where senescent nociceptors continue to release harmful SASP factors [218]. By contrast, selectively targeting senescent cells within the nociceptive lineage (source control) or dampening their secretory program (signal control) offers a mechanistic route to durable benefit [227]. From a practical perspective, this points to stage-specific treatment combinations. At first, it may be helpful to allow short-term adaptive senescence, which can protect neurons. Later on, therapy should shift toward removing long-lasting senescent nociceptors through BCL-2–dependent senolysis, blocking harmful SASP signals with JAK/STAT or p38 inhibitors or with IL-6/IL-6R blockade, and restoring balance in redox and DNA-damage pathways that keep cells locked in the senescent state. In sum, neuropathic pain can be reframed as a circuit disorder in which post-mitotic senescence of neurons and glia transitions from protective pause to pathogenic persistence—one that is mechanistically tractable if the right cellular sources and timelines are targeted. Table 3 offers a systematic overview of senescence triggers, pathways, markers, circuit consequences, and practical treatment approaches.

## 6. Nrf2 as a Central Integrator of Oxidative Stress, Inflammation, Senescence, and Neuropathic Pain

Nuclear factor erythroid 2–related factor 2 (Nrf2) has emerged as a master regulator of cellular defense, positioned at the crossroads of redox signaling, inflammation, senescence, and neuropathic pain [228,229]. Through its transcriptional control over antioxidant response elements (AREs), Nrf2 orchestrates the expression of a broad array of cytoprotective genes involved in maintaining redox homeostasis, mitochondrial function, and anti-inflammatory responses [229]. These adaptive mechanisms are critical in mitigating oxidative damage, sustaining energy metabolism, and preserving the structural and functional integrity of both neuronal and non-neuronal cells exposed to chronic stress [99].

The interplay between oxidative stress, immune activation, and cellular senescence is a defining feature of neuropathic pain and age-associated pathologies [107,230,231]. By controlling this crosstalk, Nrf2 acts as a modulator and sentinel, inhibiting detrimental inflammation, lowering glial reactivity, and postponing the onset of SASP [99,232]. Moreover, Nrf2 brings together signals from inside the cell and from the environment to control how cells respond to stress. It does this through flexible interactions with key metabolic pathways, such as mTOR, and through feedback loops that involve mitochondrial signals and autophagy [233,234,235]. This integrative role makes Nrf2 a promising therapeutic target not only for alleviating chronic pain but also for delaying degenerative processes driven by oxidative and inflammatory damage.

### 6.1. Nrf2 as a Central Regulator of Redox Homeostasis and Cellular Stress Responses

Nuclear factor erythroid 2–related factor 2 plays a pivotal role in orchestrating the transcriptional response to oxidative and electrophilic stress, acting as a master regulator of genes involved in antioxidant defense, detoxification, and cellular metabolism [229,236]. Under basal conditions, Nrf2 is sequestered in the cytoplasm through its interaction with Kelch-like ECH-associated protein 1 (Keap1), which serves as a redox-sensitive adaptor protein for Cullin 3-based E3 ubiquitin ligase complexes [236,237]. This interaction promotes the ubiquitination and proteasomal degradation of Nrf2, maintaining low steady-state levels within the cell [236,237].

**Figure 6:**
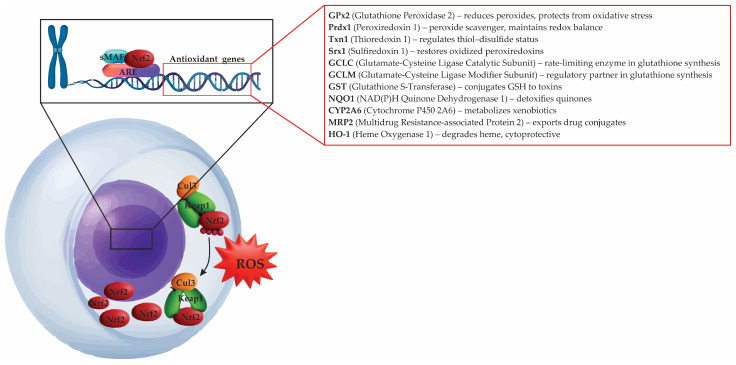
Keap1–Nrf2–ARE signaling pathway and downstream antioxidant gene activation. Under basal conditions, Nrf2 is bound to Keap1, a redox-sensitive adaptor protein that recruits Cullin 3 (Cul3)–based E3 ubiquitin ligase complexes, leading to ubiquitination and proteasomal degradation of Nrf2. Upon exposure to ROS or electrophilic stress, critical cysteine residues of Keap1 undergo covalent modifications, preventing Nrf2 degradation. Stabilized Nrf2 accumulates in the cytoplasm, translocates into the nucleus, and heterodimerizes with small Maf proteins to bind antioxidant response elements (AREs) in DNA. This induces the transcription of a wide range of cytoprotective genes, including glutathione peroxidase 2 (GPx2), peroxiredoxin 1 (Prdx1), thioredoxin 1 (Txn1), sulfiredoxin 1 (Srx1), glutamate–cysteine ligase catalytic subunit (GCLC) and modifier subunit (GCLM), glutathione S-transferases (GSTs), NAD(P)H quinone dehydrogenase 1 (NQO1), cytochrome P450 2A6 (CYP2A6), multidrug resistance–associated protein 2 (MRP2), and heme oxygenase 1 (HO-1). Collectively, these enzymes mitigate oxidative damage, detoxify xenobiotics, and maintain redox homeostasis.

Upon exposure to ROS or electrophilic agents, specific cysteine residues within Keap1 undergo covalent modifications that disrupt its ability to target Nrf2 for degradation [236,237]. As a result, Nrf2 stabilizes, accumulates in the cytoplasm, and translocates into the nucleus, where it binds to AREs in the promoter regions of its target genes. For effective ARE binding, Nrf2 forms heterodimers with small Maf proteins [236,237]. The transcriptional program activated by Nrf2 includes a broad spectrum of cytoprotective genes, notably those encoding heme oxygenase-1 (HO-1), NAD(P)H quinone oxidoreductase 1 (NQO1), glutathione S-transferases, and enzymes involved in glutathione biosynthesis [236]. HO-1, in particular, degrades heme into bioactive molecules such as carbon monoxide (CO) and bilirubin—each with potent anti-inflammatory and antioxidant properties [238]. Additionally, Nrf2 modulates inflammation by suppressing NF-κB signaling through CO-mediated inhibition and by promoting anti-inflammatory cytokine expression [239]. This process is schematically illustrated in Figure 6, which depicts the regulation of Nrf2 under basal and stress conditions, its release from Keap1, nuclear translocation, and transcriptional activation of cytoprotective target genes.

Beyond redox regulation, Nrf2 integrates signals from nutrient, metabolic, and stress pathways [240]. Its stability and activity are further modulated by autophagy-related protein p62, which binds Keap1, thereby interfering with Nrf2 degradation. This p62-mediated Nrf2 activation becomes especially relevant under impaired autophagic flux [241]. Moreover, the phosphoinositide 3-kinase/protein kinase B/glycogen synthase kinase-3 beta (PI3K/Akt/GSK-3β) signaling axis indirectly stabilizes Nrf2 by inhibiting GSK-3β–driven nuclear export and proteasomal degradation [242]. Nrf2 transcriptional activity is also enhanced by its interaction with nuclear receptors such as retinoid X receptor alpha (RXRα), which cooperatively bind to gene promoters to bolster antioxidant gene expression under stress conditions [236].

Although Nrf2 activation is generally considered cytoprotective, its dysregulation can lead to deleterious consequences. Excessive or constitutive Nrf2 activity, often driven by somatic mutations or oncogenic signaling, has been implicated in promoting tumor progression and resistance to chemotherapy by increasing antioxidant capacity, enhancing drug efflux, and suppressing apoptosis [243,244]. Chronic Nrf2 activation has also been linked to fibrosis, autoimmune pathologies, and metabolic dysfunctions, including insulin resistance and steatosis [245,246,247]. In certain cancers, upregulated Nrf2 promotes survival of malignant cells under oxidative and genotoxic stress, diminishing the efficacy of conventional therapies [243,244].

### 6.2. Nrf2-Mediated Modulation of Oxidative Stress in Neuropathic Pain

The transcription factor Nrf2 plays a central role in counteracting oxidative damage and preserving redox balance, which is essential in modulating nociceptive signaling and protecting neural structures involved in pain transmission [99]. By orchestrating the expression of a battery of antioxidant and cytoprotective genes, Nrf2 reduces the burden of ROS, thereby curbing neuronal hyperexcitability and the sensitization processes underlying neuropathic pain [228].

Within sensory pathways, Nrf2 activation enhances the transcription of enzymes such as HO-1, NQO1, SOD, catalase, and glutathione-related enzymes [236,248]. This upregulation improves the intracellular antioxidant capacity, leading to decreased nociceptor excitability and reduced glutamate-mediated excitotoxicity at synapses [99]. Nrf2 also limits the synthesis of pro-inflammatory mediators, thereby attenuating neuroinflammation—a critical component of chronic pain pathophysiology [249]. In addition to modulating redox status, Nrf2 exerts regulatory control over ion channels and neurotransmitter release, influencing the excitability of both peripheral sensory neurons and central pain circuits [250].

Neuroprotective effects of Nrf2 have been demonstrated in various experimental models. Pharmacological or genetic Nrf2 induction, especially via HO-1, was linked to strong antinociceptive effects in rodent models of inflammatory and neuropathic pain [228,251]. Agents such as cobalt protoporphyrin, curcumin, diosmetin, and sulforaphane restored antioxidant defenses, inhibited glial activation, and suppressed the release of excitatory mediators [252,253,254]. In contrast, Nrf2 inhibition, either directly or through downregulation of its downstream targets, was linked with aggravated pain behaviors and increased ROS levels [255]. Notably, a variety of treatments, such as electroacupuncture, angiotensin receptor blockade, and the administration of flavonoids, have been shown to exert analgesic effects via the Nrf2/HO-1 axis [253,256,257].

Experimental studies further demonstrated that Nrf2 is widely expressed along the nociceptive axis, including the spinal cord, DRG, and peripheral nerves [255,258,259]. Alterations in Nrf2 and HO-1 levels have been reported in various neuropathic pain models, such as those induced by paclitaxel, oxaliplatin, or sciatic nerve constriction [258,259]. These studies suggest that the redox-regulatory capacity of Nrf2 may be context-dependent, varying across disease stages, tissue compartments, and types of injury. For instance, reductions in Nrf2 and its targets have been observed in the DRG during chemotherapeutic neuropathies, whereas spinal upregulation is typical of constriction injuries [258,259].

In addition to neurons, Nrf2 also regulates the activity of glial cells, especially astrocytes and microglia, reducing their role in chronic pain by inhibiting the release of pro-inflammatory cytokines and reactive gliosis [99]. In this way, Nrf2 not only prevents oxidative and inflammatory damage to neurons but also orchestrates a broader neuroimmune regulatory network involved in pain amplification.

### 6.3. Nrf2 Signaling in Inflammation and Neuropathic Pain Modulation

The transcription factor Nrf2 has emerged as a pivotal modulator of inflammatory processes and a promising therapeutic target in neuropathic pain [99]. Its activation confers antinociceptive effects by downregulating pro-inflammatory signaling and enhancing the cellular antioxidant response [249]. In neuropathic pain models, Nrf2 activation suppresses the production of inflammatory mediators such as TNF-α and IL-1β, dampens microglial activation, and mitigates oxidative stress—factors that are central to the initiation and maintenance of pain signaling [238,239]. By attenuating the inflammatory milieu and oxidative burden within injured tissues, Nrf2 diminishes nociceptor excitability and reduces the transmission of pain impulses, while simultaneously preserving neuronal integrity and function.

Oxidative stress and inflammation are tightly interlinked through feedback amplification loops that sustain pathological pain states. For instance, reactive oxygen species promote NF-κB-mediated transcription of pro-inflammatory cytokines, which in turn exacerbate ROS generation 95]. Nrf2 activation interrupts this cycle, both by upregulating antioxidant enzymes and by inhibiting NF-κB-driven transcription [238,239]. Furthermore, Nrf2 negatively regulates the expression of cell adhesion molecules (CAMs), including vascular cell adhesion molecule 1 (VCAM-1) and E-selectin, partly through its downstream effector HO-1 [63]. This action reduces leukocyte recruitment and extravasation into sites of neural injury, thus curbing local inflammation. In addition, Nrf2 modulates the activity of matrix metalloproteinases (MMPs) and suppresses the expression of iNOS and COX-2, further dampening the inflammatory cascade [260,261].

A growing body of experimental evidence supports the therapeutic potential of targeting Nrf2 in neuropathic pain. Pharmacologic Nrf2 activators such as bardoxolone methyl have been shown to alleviate chemotherapy-induced neuropathy via suppression of inflammatory markers in the dorsal root ganglia [262]. Similarly, omaveloxolone improved mechanical allodynia and reduced neuronal apoptosis and glial activation in chronic constriction injury (CCI) models [263]. Betulinic acid and luteolin, natural compounds with known antioxidant properties, alleviated neuropathic pain by enhancing Nrf2/HO-1 signaling, suppressing glial-mediated inflammation, and modulating cytokine expression in both peripheral and central nervous tissues [264,265].

### 6.4. Nrf2–mTOR Axis at the Intersection of Oxidative Stress, Cellular Senescence, and Neuropathic Pain

Oxidative stress plays a central role in both initiating and perpetuating cellular senescence, acting upstream of SASP and shaping the inflammatory milieu that characterizes age-related pathologies and chronic pain syndromes [96]. Accumulated ROS trigger persistent DNA damage responses and activate redox-sensitive transcription factors—particularly NF-κB and C/EBPβ—that promote the expression of SASP components, including TNF-α, IL-1β, and IFN-γ [161,266]. These cytokines amplify oxidative stress in adjacent cells, forming a self-reinforcing loop that sustains chronic inflammation and senescence across tissues, a process intimately tied to the development and persistence of neuropathic pain.

Notably, recent findings have elucidated a reciprocal regulatory relationship between Nrf2 and mTOR, forming a redox-sensitive signaling hub that governs both metabolic homeostasis and senescence maintenance [233]. Nrf2 enhances mTORC1 activity through transcriptional upregulation of *mTOR* and *RagD*, a lysosome-localized GTPase essential for nutrient-sensing and mTORC1 activation [240]. Conversely, mTOR stabilizes Nrf2 by suppressing its β-TrCP–mediated ubiquitination and degradation, a Keap1-independent pathway that further ensures sustained antioxidant gene expression [240,267]. This mutual reinforcement between Nrf2 and mTOR promotes a prolonged antioxidant state that, while cytoprotective, may paradoxically support senescent cell survival and chronic low-grade inflammation, especially under conditions of persistent mTORC1 activation typical in aging tissues [268].

### 6.5. Nrf2–HCAR2 Crosstalk in Neuropathic Pain

The antioxidant transcription factor Nrf2 is analgesic in rodent neuropathic pain models, and oral dimethyl fumarate (DMF) reverses hypersensitivity via Nrf2 signaling [269]. Concurrently, the ketone-body receptor HCAR2 —activated by β-hydroxybutyrate and fumarate esters—has emerged as a modulator of neuroinflammation and pain circuitry [269,270]. Monomethyl fumarate (MMF), the active metabolite of DMF, directly engages HCAR2 on microglia/macrophages to dampen inflammatory programs, linking fumarates to this GPCR beyond their canonical Nrf2 effects [271,272]. Genetic and pharmacologic studies indicate that DMF/MMF’s protective actions in neuroinflammation require HCAR2, highlighting a dual mechanism wherein Nrf2 activation intersects with HCAR2 signaling [273].

In neuropathic pain paradigms, activating HCAR2 (via DMF or ketone pathways) alleviates allodynia and hyperalgesia, and these effects are lost in HCAR2-deficient animals, underscoring receptor involvement in nociceptive control [274,275]. Medicinal chemistry and recent reviews further position HCAR2 as a druggable anti-inflammatory/analgesic target, reinforcing the rationale for therapies that co-opt both Nrf2 and HCAR2 axes [276,277].

In the context of neuropathic pain, this crosstalk becomes pathophysiologically relevant. Excess ROS not only exacerbate mitochondrial dysfunction and glial activation but also sustain SASP-driven inflammation in dorsal root ganglia and spinal cord neurons. Nrf2 activation counters this by restoring redox balance, suppressing pro-inflammatory cytokine production, and modulating mTOR-driven metabolic reprogramming. However, in certain contexts, prolonged Nrf2–mTOR co-activation may stabilize senescent-like phenotypes in neural and glial cells, potentially contributing to pain chronification. To synthesize these interactions, we provide a concise overview in Table 4.

## 7. Conclusions

Neuropathic pain is sustained by an interconnected network of oxidative stress, neuroinflammation, mitochondrial dysfunction, and cellular senescence, in which nerve injury triggers immune activation, ion channel remodeling, and glial reactivity, while persistent redox imbalance amplifies excitability and cytokine-driven inflammation. Mitochondrial impairment fuels reactive species production and energy failure, and post-mitotic senescence of neurons and glia maintains a pro-inflammatory milieu through the senescence-associated secretory phenotype. The transcription factor Nrf2 acts as a central regulator by restoring redox homeostasis, modulating inflammation, preserving mitochondrial function, and counteracting senescence-associated signaling, with experimental evidence showing its activation alleviates neuropathic pain. Given its context-dependent effects, optimal management will require stage-specific, mechanism-based strategies that integrate redox modulation, mitochondrial support, senescence control, and neuroimmune regulation to achieve durable pain relief and improved functional outcomes.

## 8. Future Perspectives

Despite substantial advances in understanding the interplay between oxidative stress, inflammation, mitochondrial dysfunction, and cellular senescence in neuropathic pain, several critical knowledge gaps remain. First, the precise temporal dynamics and tissue specificity of these processes are incompletely mapped, limiting the ability to identify optimal therapeutic windows. The cellular sources of pathogenic signals—particularly the relative contributions of senescent nociceptors versus glial cells—require clearer delineation. Furthermore, the heterogeneity of neuropathic pain across etiologies and patient populations complicates the translation of preclinical findings into broadly effective interventions. Biomarkers capable of non-invasively detecting redox imbalance, senescence activity, and mitochondrial dysfunction in vivo are still underdeveloped, hindering patient stratification and real-time monitoring of treatment responses.

Promising therapeutic avenues include selective modulation of Nrf2 signaling to restore redox homeostasis while avoiding sustained overactivation that may stabilize senescent phenotypes. Targeted senotherapeutics—either senolytics aimed at clearing maladaptive senescent cells or senomorphics designed to suppress the senescence-associated secretory phenotype (SASP)—offer potential to dismantle the inflammatory–excitatory loop sustaining chronic pain. Mitochondria-directed interventions, such as matrix-targeted antioxidants, quality-control enhancers, and bioenergetic modulators, represent another mechanistically grounded strategy. Combination regimens pairing redox modulation with anti-inflammatory, glia-targeted, or senescence-modifying agents may yield synergistic benefits, particularly when tailored to disease stage and cellular targets.

However, several barriers impede clinical translation. Many preclinical models fail to capture the complexity and chronicity of human neuropathic pain, underscoring the need for predictive and clinically relevant experimental systems. The lack of standardized clinical endpoints for redox- and senescence-targeted therapies complicates trial design and cross-study comparison. Additionally, tissue-specific drug delivery, off-target effects, and compensatory biological responses remain major challenges for sustained efficacy. Overcoming these obstacles will require integrated, multidisciplinary efforts that combine mechanistic discovery with biomarker development, advanced modeling, and carefully designed early-phase clinical studies.

## 9. Materials and Methods

A systematic literature search was conducted in PubMed, Scopus, and Web of Science to identify peer-reviewed publications examining the interplay of oxidative stress, inflammation, mitochondrial dysfunction, cellular senescence (including SASP), and Nrf2 signaling in the pathophysiology of neuropathic pain. The search covered records from database inception to 1 September 2025.

Search strategies combined Medical Subject Headings (MeSH) and free-text keywords. Representative terms and Boolean logic included: “neuropathic pain” OR allodynia OR hyperalgesia AND (“oxidative stress” OR ROS OR RNS OR “mitochondrial dysfunction” OR mitochondria OR NOX OR “nitric oxide synthase” OR peroxynitrite) AND (senescence OR “cellular senescence” OR SASP OR “senescence-associated” OR Nrf2 OR NRF2 OR Keap1) AND (glia OR microglia OR astrocyte OR “central sensitization” OR TRPA1 OR TRPV1). Database-specific thesauri (e.g., MeSH/Emtree) were mapped to these concepts to optimize sensitivity and specificity. Reference lists of eligible articles and relevant reviews were also hand-searched to capture additional records.

Eligibility criteria

We included: (a) original experimental studies in vitro and in vivo (animal models of neuropathic pain), (b) clinical studies in humans (observational or interventional) directly probing oxidative/redox, mitochondrial, inflammatory–glial, senescence/SASP, or Nrf2-Keap1 pathways in neuropathic pain mechanisms, and (c) systematic reviews/meta-analyses that synthesized such mechanistic evidence. Studies were required to investigate at least one mechanistic axis relevant to this review (e.g., NOX/iNOS–derived oxidants, mitochondrial ROS, TRP/NaV redox gating, microglia/astrocyte-driven neuroinflammation, markers of cellular senescence, SASP mediators, or Nrf2-linked antioxidant responses).

We excluded: articles not focused on neuropathic pain; studies centered exclusively on nociception without neuropathic context; papers lacking an oxidative/mitochondrial, inflammatory–glial, senescence/SASP, or Nrf2 component; non-original content (editorials, letters, opinions), conference abstracts without full data; non-English publications; and duplicates or superseded datasets. When multiple reports drew on overlapping cohorts or experiments, the most comprehensive or most recent article was retained.

Screening process

Records from all databases were exported and deduplicated. Two reviewers independently screened titles/abstracts, then assessed full texts; disagreements were resolved by discussion with a third reviewer. Data extraction captured model/system, neuropathic-pain etiology, molecular targets (e.g., Nrf2, NF-κB, MAPK, COX-2, mitochondrial pathways, DDR, p16/p21, SASP), cell types (neurons, microglia/astrocytes, immune cells), assays/readouts (oxidative markers, cytokines, electrophysiology/behavior), and directionality of effects. Given heterogeneity, synthesis was thematic across (1) mitochondrial/redox injury and DDR; (2) glial-driven neuroinflammation; (3) senescence/SASP circuits; and (4) bidirectional crosstalk sustaining chronic neuropathic pain.

Study selection and PRISMA flow.

**Figure 7:**
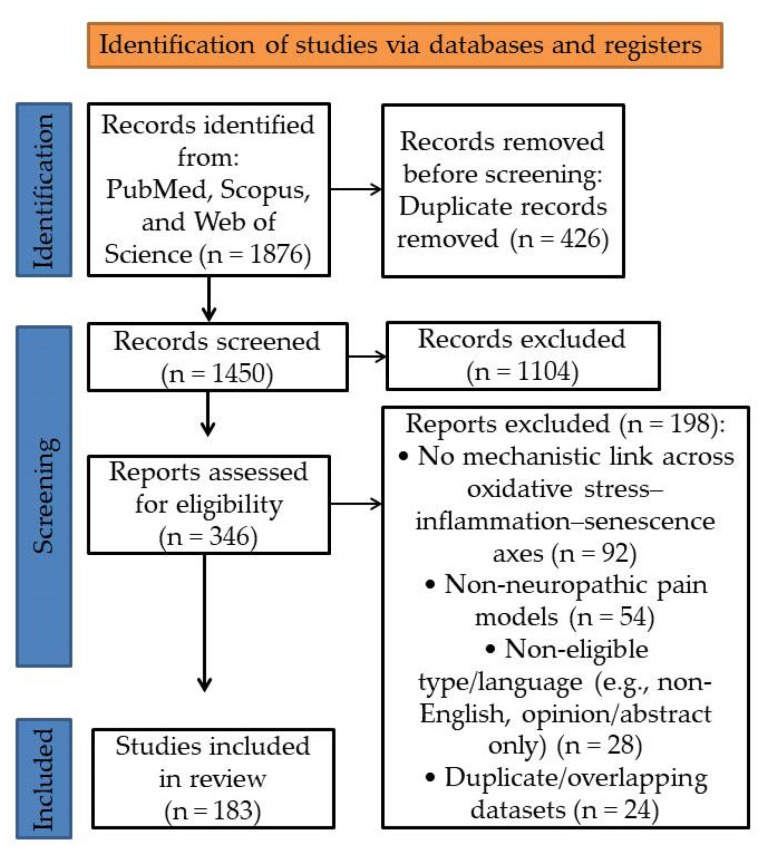
Flowchart of study selection process.

The search retrieved 1876 records. After removing 426 duplicates, 1450 records remained for title/abstract screening. 1104 were excluded as off-topic or not meeting inclusion criteria. 346 full-text articles were assessed; 198 were excluded with reasons: 92 lacked mechanistic linkage between the predefined axes, 54 addressed non-neuropathic pain, 28 were non-English or non-eligible article types, and 24 duplicated data. 148 studies met all criteria and were included in the qualitative synthesis. The selection process is depicted in Figure 7 (PRISMA 2020).

## Figures and Tables

**Table 1:** Inflammation and Neuropathic Pain—Core Mechanisms and Implications.

Mechanistic Node/Site	Core Mediators and Signals	Clinical Significance/Therapeutic Implication	References
Peripheral immune activation (lesion/DRG)	DAMPs, eicosanoids; endothelial activation → leukocyte extravasation; ROS/RNS; cytokines/chemokines	Early peripheral anti-inflammatory control to curb nociceptor hyperexcitability and prevent central amplification.	[27,47,48,49,50]
Danger sensing and inflammatory transcription	TLR4–MyD88/TRIF → NF-κB/MAPK; IL-6–gp130/JAK–STAT	Consider TLR4/NF-κB/JAK–STAT modulators to blunt sustained pro-inflammatory programs and astrocyte reactivity.	[51,52,53,54]
Prostaglandin coupling to excitability	COX-2 → PGE_2_ → EP → cAMP–PKA/PKC; ion-channel phosphorylation	Use COX-2 inhibitors and/or EP antagonists to decouple cytokine surges from synaptic potentiation and hyperexcitability.	[53,54]
Peripheral transduction plasticity	TNF/IL-1β ↑ NaV, ↓ K^+^; PGE_2_/oxidized lipids → TRPV1 sensitization, TRPA1 activation	NaV/TRP-directed strategies plus antioxidant/electrophile-quenching to reduce repetitive firing and thermal/chemical hyperalgesia.	[56,57,58]
Central glial-driven sensitization	Barrier compromise; microglia NOX2/p38 → IL-1β/TNF/BDNF → TrkB → KCC2 ↓; astrocytes STAT3/NF-κB → COX-2/PGE_2_ + chemokines	Time-staged approach: microglia targets (P2X4/P2X7, p38, NOX2) early; astrocyte/COX-2/EP/chemokine blockade for chronic maintenance.	[61,62,63,64,65,66,82,83,84,85,86,87,88,89,90,91]
Oxidative–inflammatory loop and resolution levers	NOX/iNOS → ROS/RNS; cGAS–STING and NLRP3 → IL-1β maturation; SPMs/NPD1 → GPR37; efferocytosis; M2 programming	Combine NOX/iNOS or cGAS–STING/NLRP3 inhibition with pro-resolving mediators and macrophage reprogramming to terminate inflammation.	[32,67,68,69,71,72,78,79,80]

**Table 2:** Oxidative Stress in Neuropathic Pain—Key Nodes, Effects, and Therapeutic Implications.

Mechanism/Source	Key ROS/RNS or Node	Effect on Pain Circuitry	Clinical Significance/ Therapeutic Implication	References
Mitochondrial ETC leak (Complex I/III; Fenton chemistry)	O_2_•^−^ → H_2_O_2_ → •OH	Oxidative injury, energy failure, ectopic firing	Limit reverse electron transport; optimize substrates; manage iron; use matrix-targeted antioxidants	[99,100,101,120,121,122,123,124]
NOX/iNOS pathways	NOX1/2/4, DUOX1/2; NO• → ONOO^−^	Protein nitration/DNA damage; mitochondrial enzyme inactivation	NOX/iNOS modulation; peroxynitrite handling	[103,104,105,106,123,124,125,126,127,128]
Redox gating of channels (TRPA1/TRPV1; NaV/Kv/HCN)	4-HNE, isoprostanes; S-nitrosylation/oxidation	Lowered thresholds; repetitive firing; increased gain	TRP modulators; NaV-targeted agents; antioxidant strategies	[131,132,133,134,135]
Microglial redox signaling	NOX2 → p38/JNK/NF-κB; BDNF → TrkB → ↓KCC2	Disinhibition; central sensitization/LTP-like potentiation	Early microglia-targeted therapy; TrkB/KCC2 axis (investigational)	[63,64,65,138,139,140]
Astrocyte maintenance	STAT3/NF-κB; COX-2 → PGE_2_ → EP	Glutamate dysregulation; persistent synaptic gain	COX-2 or EP antagonism; astrocyte-focused modulation	[137]
Innate immune coupling	mtDNA/oxidized DAMPs → cGAS–STING; NLRP3 inflammasome	IL-1β maturation; sustained cytokine excess	Target cGAS–STING/NLRP3 (investigational)	[141,142]
Mitochondrial integrity	Cardiolipin oxidation; impaired biogenesis/mitophagy	Supercomplex destabilization; ROS-rich, depolarized mitochondria	Cardiolipin-stabilizing peptides; support mitophagy/biogenesis	[147,148,149,150,151]

**Table 3:** Cellular Senescence in Neuropathic Pain—Triggers, Pathways, Markers, Consequences, and Therapeutic Implications.

Theme	Triggers/Drivers	Core Pathways and Markers	Key Effects in Pain Biology	Clinical Significance/Therapeutic Implication	References
Definition and types	Replicative, oncogene-, therapy-, stress-induced; MiDAS; immune-driven	p53–p21, p16–Rb; SA-β-gal; SASP	Context-dependent: repair vs. chronic inflammation	Use multi-marker panels; recognize MiDAS/SASP heterogeneity	[95,156,157,158,159,160,161,162,163,164,165,166,167,168,169,170,171,172]
DDR gatekeeping	Telomere attrition; DNA double-strand breaks	γH2AX, MDC1, 53BP1; ATM/ATR → CHK1/2 → p53 → p21; SAHF	Irreversible arrest; checkpoint enforcement	DDR biomarkers for identification; potential checkpoint modulation	[163,164,165,166,167,168,169,173,174,201]
Oxidative stress–mitochondria loop	ROS/RNS overload; mtDNA damage; ETC leak; peroxisomal insufficiency	Complex I/III ROS; catalase decline; AMPK; ATM–Akt–mTORC1–PGC-1α; glycolytic shift	ROS amplification, energy failure, Ca^2+^ dysregulation	Antioxidants/ROS scavenging; mito-support (biogenesis, mitophagy, fission–fusion balance)	[95,148,184,185,186,187,188,189,190,191,192,193,194,195,196]
SASP control and propagation	Persistent stress/DDR; redox crosstalk	NF-κB, p38 MAPK, mTOR, C/EBPβ; cGAS–STING; IL-1β/IL-6/IL-8, chemokines, MMPs, EVs	Paracrine senescence; tissue remodeling; chronic inflammation	Target SASP nodes (NF-κB/p38/mTOR; cGAS–STING); time-sensitive modulation	[95,161,165,197,198]
Neural and glial senescence (PoMiCS)	Axotomy, chemo, oxidative/genotoxic stress; aging	Neurons/glia with p21/p16, SA-β-gal, γH2AX; TRPV1^+^ DRG IL-6 SASP; astrocyte/microglia SASP	Altered excitability, disinhibition, central sensitization	Stage-aware therapy: nociceptor source control + glial signal control (e.g., IL-6/JAK–STAT, COX-2/EP, chemokines)	[213,214,215,216,217,218,219,220,221,222,223,224,225]

**Table 4:** Nrf2 as a Central Integrator of Oxidative Stress, Inflammation, Senescence, and Neuropathic Pain.

Module/Axis	Core Mechanisms and Evidence	Clinical Significance/Therapeutic Implication	References
Keap1–Nrf2–ARE core	Oxidant/electrophile modification of Keap1 cysteines → Nrf2 stabilization → nuclear translocation; sMaf heterodimers bind ARE; induction of HO-1, NQO1, GSTs, GSH biosynthesis enzymes; HO-1 generates CO/bilirubin (anti-inflammatory/antioxidant).	Boosts cellular redox buffering, preserves mitochondria, limits excitotoxicity; HO-1/CO axis provides analgesic anti-inflammatory effects.	[228,235,236,237]
Anti-inflammatory crosstalk	Nrf2 restrains NF-κB; lowers CAMs (VCAM-1, E-selectin); suppresses iNOS, COX-2, MMPs.	Reduces leukocyte recruitment and neuroinflammation; complements standard analgesics; candidate for disease-modifying strategies.	[63,237,238,259,260]
Neuro-glial modulation	Nrf2 decreases microglial activation and astrocyte reactivity; dampens TNF/IL-1β; limits glutamate-driven excitability; influences ion channels/neurotransmission.	Attenuates central sensitization; pairs well with glia-targeted and synaptic-modulating therapies.	[98,238,239,240,241,242,243,244,245,246,247,248,249]
Evidence in pain models and activators	Nrf2/HO-1 induction yields antinociception; agents: cobalt protoporphyrin, curcumin, diosmetin, sulforaphane; bardoxolone, omaveloxolone; electroacupuncture/ARBs/flavonoids act via Nrf2; Nrf2 inhibition worsens pain/ROS.	Pharmacologic Nrf2 activation is analgesic across models; translational potential for chemotherapy- and injury-induced neuropathies.	[227,250,251,252,253,254,255,256,261,262,263,264]
Integrative signaling nodes	p62 competes for Keap1 (autophagy link); PI3K/Akt inhibits GSK-3β → stabilizes Nrf2; cooperation with RXRα.	Opportunity to combine Nrf2 activation with autophagy support or PI3K/Akt tuning; context-aware dosing.	[235,240,241]
Nrf2–mTOR and senescence	Reciprocal Nrf2 ↔ mTOR reinforcement (Nrf2 ↑ mTOR/RagD; mTOR ↓ β-TrCP-mediated Nrf2 degradation); impacts SASP and persistence of senescent phenotypes.	Stage-specific use: activate Nrf2 to quell ROS/inflammation, but avoid chronic overactivation that may stabilize senescence; consider mTOR modulation.	[232,239,266,267]
Localization and dynamics in pain axis	Nrf2/HO-1 expressed in spinal cord, DRG, peripheral nerves; chemo-neuropathy vs. CCI show compartment- and stage-dependent shifts.	Target tissues/stages selectively; consider biomarkers of Nrf2 activity for patient stratification.	[63,254,257,258,259,260,261,262,263,264,265,266,267,268,269,270,271,272,273,274,275,276,277]
Risks of chronic/constitutive activation	Tumor promotion, therapy resistance; fibrosis, autoimmunity; metabolic dysregulation (insulin resistance, steatosis).	Careful patient selection/monitoring; caution in active malignancy; balance benefit vs. risk.	

## Data Availability

As this is a review article, no new data were created or analyzed. All data supporting the findings of this study are available in the cited literature. Therefore, a data availability statement is not applicable.

## Abbreviations

The following abbreviations are used in this manuscript:

4-HNE	4-hydroxynonenal
53BP1	p53-binding protein 1
AMPK	AMP-activated protein kinase
AREs	antioxidant response elements
ATM	ataxia-telangiectasia mutated
ATP	adenosine triphosphate
ATPSc-KMT	ATP synthase c-subunit lysine N-methyltransferase
ATR	ataxia-telangiectasia and Rad3-related
BCL-2	B-cell lymphoma 2
BDNF	brain-derived neurotrophic factor
BrdU	bromodeoxyuridine
C/EBPβ	CCAAT/enhancer-binding protein beta
CCI	chronic constriction injury
CCR2	C-C chemokine receptor type 2
CCL11	C-C motif chemokine ligand 11
CCL2	C-C motif chemokine ligand 2
CGRP	calcitonin gene-related peptide
CHK1	checkpoint kinase 1
CHK2	checkpoint kinase 2
CO	carbon monoxide
CAMs	cell adhesion molecules
CDKs	cyclin-dependent kinases
COX-2	cyclo-oxygenase-2
CSF1	colony-stimulating factor 1
CX3CL1	C-X3-C motif chemokine ligand 1
CX3CL1–CX3CR1	C-X3-C motif chemokine ligand 1–C-X3-C chemokine receptor 1
CXCL1	C-X-C motif chemokine ligand 1
DAMPs	danger-associated molecular patterns
DDR	DNA damage response
DRG	dorsal root ganglia
DRP1	dynamin-related protein 1
Duox	dual oxidases
E2F	E2 promoter binding factor
EGF	epidermal growth factor
EP	E-type prostanoid
ER	endoplasmic reticulum
ERK	extracellular signal-regulated kinase
ERO1–PDI	endoplasmic reticulum oxidoreductin 1–protein disulfide isomerase
ETC	electron transport chain
Fe^2+^	ferrous iron
GABA	gamma-aminobutyric acid
GADD45A	growth arrest and DNA damage-inducible alpha
GDF15	growth differentiation factor 15
GPR37	G protein-coupled receptor 37
GRB2	growth factor receptor-bound protein 2
GSH	glutathione
GluN1	glutamate ionotropic receptor NMDA type subunit 1
GM-CSF	granulocyte-macrophage colony-stimulating factor
gp130	glycoprotein 130
H_2_O_2_	hydrogen peroxide
HCN	hyperpolarization-activated cyclic nucleotide-gated
HMGB1	high-mobility group box 1
HO-1	heme oxygenase-1
HuR	human antigen R
IASP	International Association for the Study of Pain
IFN-γ	interferon-gamma
IGF-1	insulin-like growth factor 1
IL	interleukin
IL-1β	interleukin-1β
IL-4	interleukin-4
IL-10	interleukin-10
IL-17	interleukin-17
IL-33	interleukin-33
iNOS	inducible nitric oxide synthase
IP_3_R	inositol 1,4,5-trisphosphate receptor
IRF	interferon regulatory factor
JAK–STAT	Janus kinase–signal transducer and activator of transcription
JNK	c-Jun N-terminal kinase
K^+^	potassium
KCC2	potassium-chloride cotransporter 2
Kv	voltage-gated potassium
LCN2	lipocalin-2
LPS	lipopolysaccharide
MAMs	mitochondria-associated membranes
M-CSF	macrophage colony-stimulating factor
MAPK	mitogen-activated protein kinase
MDC1	mediator of DNA damage checkpoint protein 1
MFN1/2	mitofusin-1/2
MiDAS	Mitochondrial dysfunction–associated senescence
MMPs	matrix metalloproteinases
mPTP	mitochondrial permeability transition pore
mtDNA	Mitochondrial DNA
mTOR	mechanistic target of rapamycin
mTORC1	mechanistic target of rapamycin complex 1
MyD88	myeloid differentiation primary response 88
NAD^+^	nicotinamide adenine dinucleotide
NCLX	sodium/calcium exchanger
NF-κB	nuclear factor kappa-light-chain-enhancer of activated B cells
NGF	nerve growth factor
NLRP3	NOD-, LRR-, and pyrin domain-containing protein 3
NMDA	N-methyl-D-aspartate
NO	nitric oxide
NOX2	NADPH oxidase 2
NPD1	neuroprotectin D1
NRF1/2	nuclear respiratory factors 1 and 2
Nrf2	nuclear factor erythroid 2–related factor 2
NQO1	NAD(P)H quinone oxidoreductase 1
O_2_•^−^	superoxide anion
ONOO^−^	peroxynitrite
OPA1	optic atrophy protein 1
P2X4	P2X purinoceptor 4
P2X7	P2X purinoceptor 7
PCNA	proliferating cell nuclear antigen
PGC-1α	peroxisome proliferator-activated receptor gamma coactivator 1-alpha
PGE2	prostaglandin E2
PI3K	phosphoinositide 3-kinase
PINK1	PTEN-induced kinase 1
PoMiCS	post-mitotic cellular senescence
PRRs	Pattern-recognition receptors
p53	protein p53
RAGE	receptor for advanced glycation end products
Rb	retinoblastoma
Ref-1	redox factor-1
ROS/RNS	reactive oxygen and nitrogen species
SA-β-gal	senescence-associated β-galactosidase
SAHF	senescence-associated heterochromatic foci
SASP	senescence-associated secretory phenotype
SOD2	superoxide dismutase 2
ST2	suppression of tumorigenicity 2
TGF-β	transforming growth factor-beta
TFAM	mitochondrial transcription factor A
thermo-TRPs	Thermo-transient receptor potential channels
Th	T-helper
TLR4	Toll-like receptor 4
TNF-α	tumor necrosis factor-alpha
TrkB	tropomyosin receptor kinase B
TRIF	TIR-domain-containing adapter-inducing interferon-β
TRPA1	transient receptor potential ankyrin 1
TRPV1	transient receptor potential vanilloid 1
TRPV4	transient receptor potential vanilloid 4
TSP1	thrombospondin-1
UV	Ultraviolet
VCAM-1	vascular cell adhesion molecule 1
WNT	Wingless/Integrated
cAMP–PKA/PKC	cyclic adenosine monophosphate–protein kinase A/protein kinase C
cGAS–STING	cyclic GMP–AMP synthase–stimulator of interferon genes
NaV	voltage-gated sodium channels
